# Deep learning-based EV battery fault diagnosis using RBBMO with CAR-TATNET detection approach

**DOI:** 10.3389/frai.2026.1876996

**Published:** 2026-09-10

**Authors:** G. Tamizharasi, G. K. Rajini

**Affiliations:** School of Electrical Engineering, VIT University, Vellore, Tamil Nadu, India

**Keywords:** context-aware recurrent neural network with adaptive temporal transformer, deep learning, electric vehicle, improved complete ensemble empirical mode decomposition with adaptive noise, red-billed blue magpie optimization, short-time Fourier transform

## Abstract

Ensuring the safety and dependability of power batteries has become a major concern due to the rapid expansion of electric vehicles (EVs), and fault detection has emerged as an essential approach for guaranteeing system stability. This study develops a Deep Learning (DL)-based defect prediction technique that combines an optimization algorithm and a Context-Aware Recurrent Neural Network with Adaptive Temporal Transformer (CAR-TATNet) to overcome these drawbacks. The aim is to reduce noise and improve the extraction of pertinent operating modes from EV battery signals. The dataset was first pre-processed by data cleaning and with Improved Complete Ensemble Empirical Mode Decomposition with Adaptive Noise (ICEEMDAN). Key time-frequency features linked to EV battery fault patterns were captured using Short-Time Fourier Transform (STFT). Inspired by the way magpies forage, the Red-Billed Blue Magpie Optimization (RBBMO) algorithm finds the most important traits for effective feature selection. Finally, the CAR-TATNet model was employed to model long-term dependencies in temporal sequences and adaptively respond to time-varying fault patterns. The developed methodology performs better than current methods, according to experimental validation in Python software, with a superior accuracy of 94.94%, providing a potential alternative for precise EV battery fault detection.

## 1 Introduction

One promising way to decrease dependence on fossil fuels and accelerate efforts to become carbon neutral is through the electrification of transportation. In order to achieve this, however, electric vehicle (EV) batteries must function well and be reasonably priced. The goal of becoming carbon neutral by 2050 has drawn a lot of attention to vehicle electrification. Because of their high-power density, energy density, lengthy lifetime, and comparatively short self-discharge rates, Lithium-ion batteries (LIBs) are frequently employed in EV applications (Kavin and Karuvelam, 2023). However, battery-related incidents involving EVs are occurring worldwide, endangering lives and property. Small battery malfunctions are difficult to identify when EVs are in operation, and prolonged use raises the possibility of producing additional heat, which results in thermal runaway (Zhao et al., 2025; Vidyabharathi et al., 2023). Battery performance is hampered by these defects, and the risks worsen with further use, resulting in catastrophic failures. Modifying the lithium-ion battery’s architecture and structure quickly to increase safety is challenging (Karthick, 2025).

Researchers are urged to emphasize defect recognition and diagnosis following multiple electric-vehicle incidents caused by LIB system failures. Battery management systems (BMS) have some basic criteria built in to identify problems, but they are not very reliable (Wu et al., 2022). As a result, there are pressing issues that need to be resolved, such as the prompt and precise identification of battery problems and the delivery of useful early warnings (Zhang et al., 2024; Khaneghah et al., 2023). Researchers have created a number of techniques to accomplish accurate battery failure detection. Because Deep Learning (DL) is better at processing complicated, high-dimensional data, recent developments in the field have shown impressive performance in battery problem detection. Noise reduction is naturally aided by the signal decomposition approach (Li et al., 2024). More robust features are typically produced by identifying and eliminating irrelevant or high-frequency noise components prior to feature extraction. Subjectivity is introduced by poor decisions that have a substantial effect on the quality of the decomposition and the resulting characteristics. By performing simultaneous variable or feature selection, sparse principal component analysis (SPCA) (Li et al., 2022) eliminates noise and redundant information from unimportant variables. Certain statistical analyses and visualizations are complicated by correlations between SPCA components. By encoding signals with fewer significant coefficients, the Wavelet transform (Kosuru and Kavasseri Venkitaraman, 2023) efficiently compresses data, lowering processing and storage requirements. Nevertheless, it is computationally demanding, particularly for high-dimensional data or real-time applications, even if it is frequently quicker than intricate numerical models. Since noise tends to be dispersed among numerous small-magnitude coefficients, the Discrete wavelet transform (DWT) (Chakraborty et al., 2025) method effectively separates noise from the actual signal components by thresholding wavelet coefficients. Wavelet theory knowledge is necessary to select the appropriate wavelet basis functions and properly understand the resulting coefficients. Thus, the Short-Time Fourier Transform (STFT) is utilized to extract the most pertinent feature from the pre-processed signal.

Factor Analysis (Liu et al., 2024) greatly reduces the dimensionality of the dataset without sacrificing too much information by efficiently condensing a large number of correlated variables into a smaller collection of uncorrelated factors. It is more complicated than more straightforward feature selection techniques and its proper implementation and interpretation call for a deeper grasp of statistics. Relevant features are preserved while superfluous features are effectively removed by the Maximal Relevance and Minimal Redundancy (MRMR) (Wang et al., 2023) method. For complicated continuous data or particular learning paradigms like federated learning, MRMR needs to be adjusted. To maximize the significance of features, DE is paired with the distance discriminant (FSDD) (Selvaraj et al., 2024). Following signal analysis, the Hall signal’s characteristics either lower the classifier’s recognition rate or have no effect on the recognition outcome through feature selection and deletion, which lowers the recognition system’s computation costs. FSDD is not equipped to capture complicated relationships or dependencies among features, which are crucial for classification performance, because it assesses features either separately or in a limited way. For the investigation of non-linear and non-stationary signals, an enhanced Hilbert–Huang Transform (HHT) (Lee and Hung, 2021) method is very useful. The iterative nature of the EMD process and the numerous alternatives put out to address its shortcomings make it difficult to implement successfully. Thus, the Red-Billed Blue Magpie Optimization (RBBMO) is utilized for feature selection in the current research. DL is best suited for fault diagnosis in EV batteries.

## 2 Literature review

A hybrid Convolutional Neural Networks (CNN)-Bidirectional Long Short-Term Memory (BiLSTM) approach (Nsaif et al., 2022) has improved prediction accuracy and stability by combining the deep features of 1D CNN mining data with the time series analysis capabilities of BiLSTM. Without enough regularization, model interpretability is hampered and the risk of overfitting increases, necessitating a large amount of data and adjustment. Generative Adversarial Network (GAN)-CNN-LSTM (Gao et al., 2023) automatically learns both spatial and temporal features to detect whether a battery failure has arisen. To balance the dataset, the GAN module learns the distribution of a small number of real fault cases and produces high-quality synthetic fault samples. The CNN-LSTM module preserves the original structure and facilitates effective classification by directly modelling multivariate time-series input without the requirement for flattening. Deep CNN/LSTM layers and the adversarial nature of GANs result in extremely long training and convergence times. By automatically learning pertinent features, the CNN-Bidirectional Gated Recurrent Unit (BiGRU) model (Liu et al., 2025) reduces the need for manual data preparation. It works well for traffic flow, energy load series, and multi-sensor data when both temporal and spatial patterns are important. If training data is not adequately regularized, the deep, complicated nature results in memorization, particularly with smaller datasets.

The CNN Patch Transformer model (Mishra et al., 2025) combines the Transformer’s ability to represent long-range dependencies and global context with the benefits of CNNs’ local feature extraction. One disadvantage is the architectural complexity of combining two distinct neural network types. It is difficult to integrate the parts and fuse features. The multidimensional battery voltage parameters are effectively predicted by the GRU-LSTM (Li et al., 2025) model. In addition to increasing voltage forecast accuracy, the GRU-LSTM model’s integration with BMS offers more precise decision care for battery health management. An incorporation of GRU-LSTM in real-time BMS of EVs to confirm dependability and performance under challenging operating situations needs to be considered. GBDT-iForest-Boxplot (Zhang et al., 2025), a power battery anomaly prediction technique, is created to get around the drawbacks of conventional fault detection methods. It offers strong assistance for the enhancement and optimization of EV protection warning systems by resolving the drawbacks of conventional techniques, taking into account numerous states and multidimensional elements and highlighting practical application needs. The multi-model fusion approach’s computational efficiency poses a problem for real-time implementation in large-scale battery energy storage devices, which demand significant computing power.

By merging transformer-based learning and persistent homology, topological DL (Batlapadu and Ansari, 2024) is utilized to gain a deeper understanding of battery health. By combining spatial and temporal signals, this combined framework aids in the early detection of errors and the monitoring of performance variations over time. A large amount of computing resources are needed to syndicate Topological Data Analysis (TDA) with self-attention mechanisms in transformers. Complex, non-linear relationships in high-dimensional data are modelled by the reconstruction-based model with the transformer and LSTM model (Wang et al., 2023), which is also very scalable. Models are too good at reconstructing data; they even learn to reconstruct abnormalities. Missed detections occur when the reconstruction error lessens owing to the model’s adaptation to anomalous inputs. Thus, the Context-Aware Recurrent Neural Network with Adaptive Temporal Transformer (CAR-TATNet) is developed in this research for detecting fault EV battery.

### 2.1 Novelty

The combination of RBBMO-feature optimization with a CAR-TATNet for EV battery issue diagnostics means the developed framework is innovative. CAR-TATNet comprises a context-aware recurrent module that continues sequential battery degradation patterns and operational dynamics across time, in contrast to traditional transformer models that mainly rely on self-attention techniques to capture global dependencies. By dynamically concentrating on vital temporal segments connected to Normal, Warning, and Fault states, rather than treating every time step equally, the adaptive temporal transformer enhances fault classification even further. Moreover, only the most informative fault-related features are supplied to the network owing to the combination of Improved Complete Ensemble Empirical Mode Decomposition with Adaptive Noise (ICEEMDAN), STFT, and RBBMO. It raises the model’s resilience in noisy operating environments, decreases feature redundancy, and enhances computational efficiency. Thus, CAR-TATNet uses contextual recurrence, adaptive temporal attention, and optimization-guided feature learning in tandem to diagnose EV battery faults more precisely and consistently than preceding transformer-based approaches.

The main objectives are

- To use ICEEMDAN to remove noise in pre-processing.
- To extract time-frequency features from the EV battery data using STFT, which provides both time and frequency domain information.
- To optimize the diagnosis process with RBBMO by effectively choosing the most pertinent features from the vast collection of extracted time-frequency information.
- To describe the temporal dependencies in EV battery data using CAR-TATNet, enhancing the robustness of fault detection.

## 3 Proposed methodology

**Figure 1:**
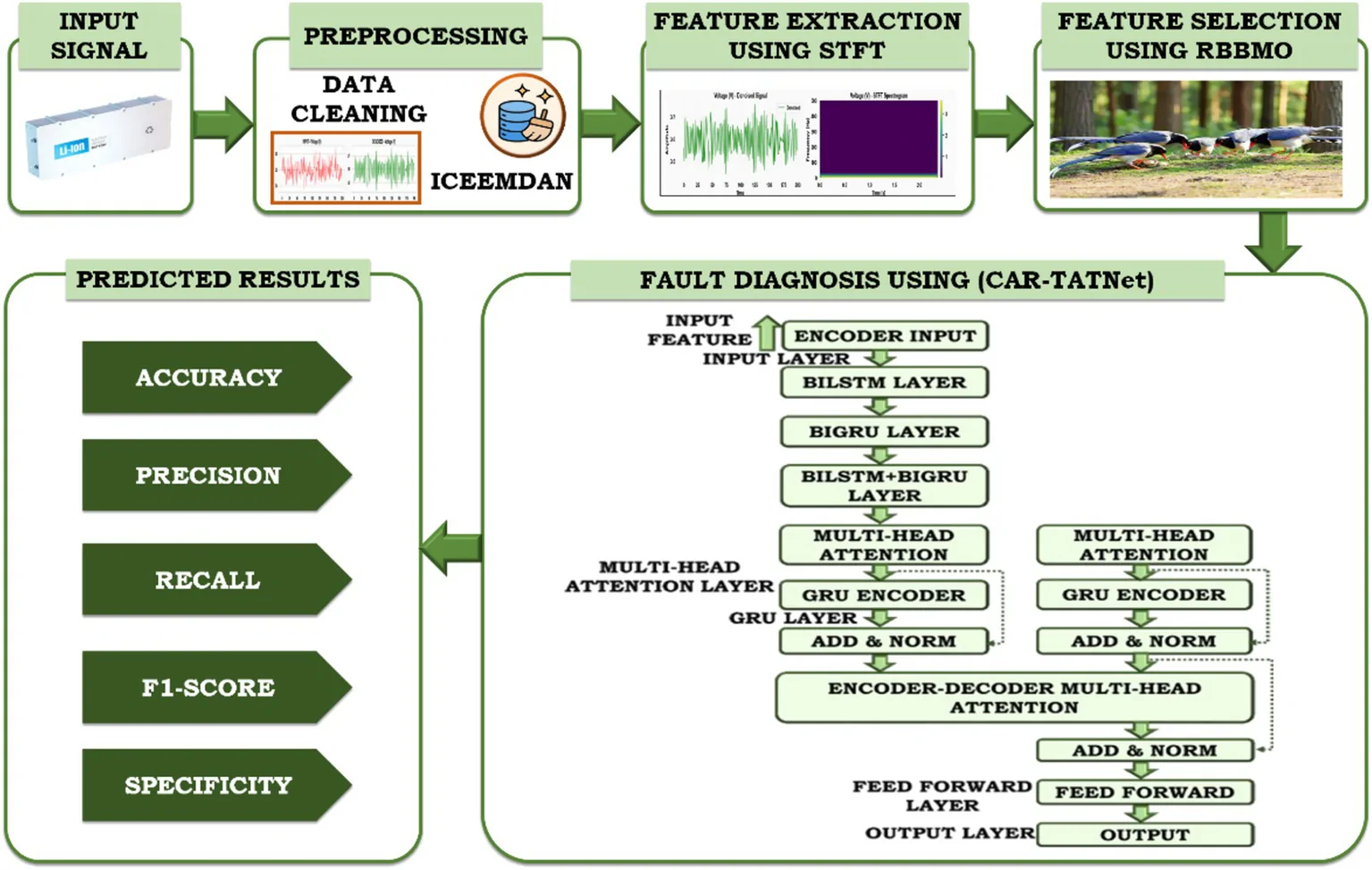
Block diagram of EV battery diagnosis.

The process begins with preprocessing, which includes ICEEMDAN to break down the raw battery signals into intrinsic mode functions after data cleaning to eliminate noise and unnecessary information, as seen in Figure 1. By separating noise and maintaining the battery’s actual operating patterns, it improves the extraction of essential data. The STFT, which performs a time-frequency analysis of signals, is then applied to the processed data. By capturing both frequency domain and time domain features, this transformation enables the model to identify dynamic fault patterns that change over time. RBBMO is then used for feature selection that effectively chooses the most pertinent characteristics from the collected time-frequency data, improving the feature set by removing unnecessary and redundant information.

This process is inspired by the way magpies forage. The chosen features are then loaded into CAR-TATNet, which combines Adaptive Temporal Transformer and Context-Aware Recurrent Neural Network (RNN). While the Adaptive Temporal Transformer models long-term dependencies and time-varying failure patterns, the RNN captures short-term temporal dependencies in the data. By learning both short-term and long-term trends in the EV battery’s performance, this hybrid technique allows the model to adaptively identify defects. By enhancing data quality, maximizing feature selection, and utilizing cutting-edge DL algorithms for accurate fault detection, these components function together to guarantee an effective and exact fault diagnosis system.

### 3.1 Preprocessing

The primary goal of data pre-processing is to find errors and prepare the collected data for denoising, slicing, and reconstruction. Data cleaning and data noise reduction are two primary mechanisms of data preprocessing. The primary goals of data cleaning and noise reduction are to address isolated instances of data loss and real discrepancies. Prior to data processing, sampling points with a 1 interval is employed in place of the original data for suitability.

#### 3.1.1 Data cleaning

Data cleansing is necessary since failures and exceptions in data collecting are unavoidable during mass transmission. Initially, the gathered battery data is analyzed to predict any missing values caused by sensor malfunctions or communication disruptions. To preserve data continuity, these missing samples are estimated by linear interpolation based on nearby observations. To evade redundancy, duplicate entries generated during repeated data logging are then predicted by timestamp verification and eliminated. In order to guarantee data consistency and dependability, numerical errors and atypical measurements are found utilizing statistical outlier detection techniques and either fixed or eliminated. After cleaning, the data is forwarded to the noise reduction phase for additional processing.

#### 3.1.2 ICEEMDAN

Following data cleaning, noise reduction addresses remaining noise in the data, such as random deviations, errors, and unexpected outliers. ICEEMDAN is used for noise reduction since noise in the data compromises the model’s resilience and accuracy. Gaussian white noise is applied to the $k^{\mathrm{th}}$ component, as opposed to the CEEMDAN which adds this noise directly into the original signal. The first stage of the Empirical Mode Decomposition determines the $k^{\mathrm{th}}$ component, which is known as Intrinsic Mode Function (IMF). Every level of the multistage decomposition procedure generates a residual term and a new IMF component. These elements are further broken down until only the residual word is left, at which point it cannot be broken down any further. When the residual term fulfils the monotonicity criteria and its amplitude stays below a predetermined threshold, the decomposition is deemed complete. ICEEMDAN modifies the accumulation of white noise in each round of the multi-level decomposition of the original signal based on the features of the current signal and the outcomes of the preceding round. ICEEMDAN’s noise addition technique allows it to avoid over-decomposition or under-decomposition scenarios, accurately detect signal termination conditions, and manage the different signal features seen in explosive vibration signals. M(·) is the original signal’s local mean value acquired by EMD, and Ek(·) is the function that builds the EMD decomposition model. The corresponding formulations are provided in Equations (1–8)

$$X^{\left(i\right)}=X+\beta_{0}E_{1}(W^{\left(i\right)})$$ (1)

The original signal is represented by X, the additional Gaussian white noise’s standard deviation is represented by $\beta_{0}$ , Gaussian white noise with a mean and variance of zero is represented by $W^{\left(i\right)}$ , and the function for determining the IMF1 is represented by $E_{1}(.)$ .

The first residual value is,

$$R_{1}=\frac{1}{N}\sum_{i=1}^{N}M(X^{\left(i\right)})$$ (2)

Where, the number of sample point is N.

The $\;k^{\mathrm{th}}$ modal component generated by the ICEEMDAN break process is denoted by k. This leads to the extraction of the first mode at k=1.

$$F_{1}=\frac{1}{N}\sum_{i=1}^{N}E_{1}(x)=X-R_{1}$$ (3)

When k is 2, the 2nd residual is obtained by using the Empirical Mode Decomposition (EMD) method to calculate the local mean of $R_{1}$ after Gaussian white noise is added.

$$R_{2}=\frac{1}{N}\sum_{i=1}^{N}M(R_{1}+\beta_{1}E_{2}(W^{\left(i\right)}))$$ (4)

Finding the difference between $R_{1}$ and $R_{2}$ yields the second modal component:

$$F_{2}=R_{1}-R_{2}=R_{1}-M(R_{1}+\beta_{1}E_{2}(W^{\left(i\right)}))$$ (5)

$k^{\mathrm{th}}\text{mode}\;$ and $k^{\mathrm{th}}$ residual are computed when k equals 3,4,…,K .

$$R_{k}=\frac{1}{N}\sum_{i=1}^{N}M(R_{k-1}+\beta_{k-1}E_{k}(W^{\left(i\right)}))$$ (6)

$$F_{k}=R_{k-1}-R_{k}$$ (7)

To extract all IMFs, the process is repeated. This keeps going until the last residual, $R_{n}$ , stops being decomposable and shows monotonic behavior. As a result, the initial signal is divided into two independent halves:

$$X=R_{n}+\sum_{i=1}^{N}F_{i}$$ (8)

Thus, it lessens the effect of outside interference and improves model performance by guaranteeing that the processed data is cleaner and more indicative of the battery’s actual operating behavior. It offers a high-quality dataset that enables more precise fault diagnosis and feature extraction.

### 3.2 STFT based feature extraction

STFT allows the model to extract the features in the EV battery by capturing both temporal and spectral properties. A Fourier transform carried out in consecutive frames is known as the STFT of a given frame s(m,n), . The related expressions are provided in Equations (9–19).

$$S(m,k)=\sum_{n}s(m,n)e^{-j2\mathrm{\pi nk}/N}$$ (9)

s(m,n)=s(n)ω(n−mL) (10)

The windowing function for *N* samples is *w*(*n*). This function is found at mL , while L is the sample shift-time step. N is the number of discrete frequencies, which is often a power-of −2 when utilizing Fast Fourier transform (FFT). The ratio of overlap among consecutive frames is (N−L)/N . Next, the Power spectrum density (PSD) is calculated:

$$P_{s}(m,k)=\frac{1}{N}{|S(m,k)|}^{2}$$ (11)

Every windowed segment at the sampling frequency $f_{s}$ is signified by N-points PSD that span the frequency $[-\frac{f_{s}}{2},\frac{f_{s}}{2}].$ Because the power spectrum is symmetric, only N/2 discrete frequencies adequately characterize it. A discrete frequency $f_{k}=\frac{kf_{s}}{N},$ where 0≤k<N/2, is represented by each frequency index k. Henceforth, it improves the overall accuracy of fault detection by strengthening the model’s capacity to predict errors across a range of operational circumstances.

### 3.3 RBBMO-based feature selection

In EV battery fault diagnosis, the RBBMO algorithm is employed for feature selection and optimizing the selection of the most relevant features from the extracted signals to enhance the fault prediction accuracy. The process is initiated with population initialization, where candidate solutions are produced within the feature space’s constraints. The population matrix comprises n candidate solutions with the number of dimensions related to the feature space of EV battery data.

$$X=[\begin{array}{ccc} x_{1,1} & \cdots & x_{1,j}\;\;x_{1,\dim-1}\;\;x_{1,\dim}\\ x_{2,1} & \cdots & x_{2,j}\;\cdots \;\;\;x_{2,\dim}\\ \begin{array}{c} \cdots \\ \vdots \\ \begin{array}{c} x_{n-1,1}\\ x_{n,1} \end{array} \end{array} & \begin{array}{c} \cdots \\ \vdots \\ \begin{array}{c} \cdots \\ \cdots \end{array} \end{array} & \begin{array}{ccc} \begin{array}{c} x_{i,j}\\ \vdots \\ \begin{array}{c} x_{n-1,j}\\ x_{n,j} \end{array} \end{array} & \begin{array}{c} \cdots \\ \vdots \\ \begin{array}{c} \cdots \\ x_{n,\dim-1} \end{array} \end{array} & \begin{array}{c} \cdots \\ \vdots \\ \begin{array}{c} x_{n-1,\dim}\\ x_{n,\dim} \end{array} \end{array} \end{array} \end{array}]$$ (12)

Where the population of candidate solution is X , dimension of feature space is dim, and population size is n. Each individual search agent is produced within the specified bound as

$$x_{i,j}=(\mathrm{ub}-\mathrm{lb})\times \mathrm{Ran}d_{1}+\mathrm{lb}$$ (13)

**Figure 2:**
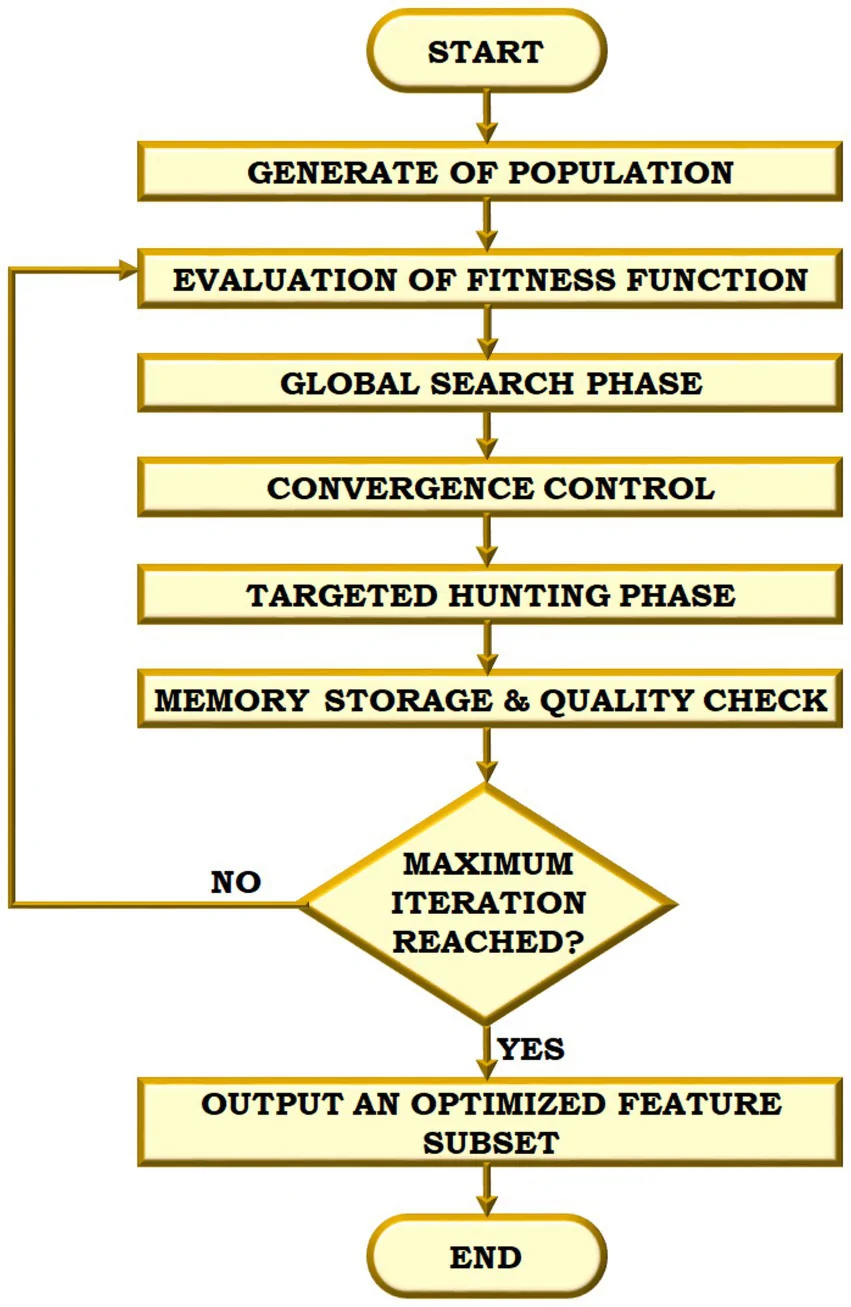
Flowchart of feature selection.

Here, lower and upper bounds are lbandub . This population then evolves via the optimization process, and the agents are iteratively updated to choose the most pertinent features based on the fitness function for fault diagnosis. In the RBBMO algorithm, the search for food imitates the behavior of red-billed blue magpies, which often hunt in groups based on environmental scenarios. In the small group search, each group dynamically searches for food resources by altering their position according to the present food source and the movements of other group members. This is impacted by the historical position and by random selection of other individuals. The flowchart of this feature section is revealed in Figure 2,

$$X^{i}(t+1)=X^{i}(t)+(\frac{1}{p}\times \sum_{m=1}^{p}X^{m}(t)-X^{\mathrm{rs}}(t))\times \mathrm{Ran}d_{2}$$ (14)

Where the randomly selected individual’s location from the population is $X^{m}(t),$ the number of magpies in a small group is p, and updated position is $X^{i}(t+1).$ When searching in larger clusters or groups, the group number varies from 10 to n individuals. In a cluster search, the agents explore food sources based on the positions of extensive range.

$$X^{i}(t+1)=X^{i}(t)+(\frac{1}{q}\times \sum_{m=1}^{q}X^{m}(t)-X^{\mathrm{rs}}(t))\times \mathrm{Ran}d_{3}$$ (15)

Where the number of search agents are q . Based on prey size and the hunting strategy, the magpies adopt distinct approaches to protect their food. The hunting behavior improves the optimization process by simulating the attack phase while the magpies alter their positions based on their proximity to food source. To enhance the overall solution to the issue, it mirrors the attack behavior and refines the search agent’s position. In the small group hunting strategy, magpies focus on smaller prey, and its approach is quick and cooperative with each agent dynamically adjusting its position according to the food source and the position of other agents in the group.

$$X^{i}(t+1)=X^{\text{food}}(t)+\mathrm{CF}\times (\frac{1}{p}\times \sum_{m=1}^{p}X^{m}(t)-X^{i}(t))\times \mathrm{Ran}d_{1}$$ (16)

$$X^{i}(t+1)=X^{\text{food}}(t)+\mathrm{CF}\times (\frac{1}{q}\times \sum_{m=1}^{q}X^{m}(t)-X^{i}(t))\times \mathrm{Ran}d_{2}$$ (17)

$$\mathrm{CF}={\left(1-(\frac{t}{T})\right)}^{\left(2\times \frac{t}{T}\right)}$$ (18)

Where the position of food is $X^{\text{food}}(t)$ and the convergence factor is CF . Food storage ensures that the magpies have a steady food supply during periods of scarcity, particularly in fluctuating environmental conditions. It serves as a mechanism for exploitation and protection of best solutions.

$$X^{i}(t+1)=\{\begin{array}{c} X^{i}(t)\;\;\;\text{if fitnes}s_{\mathrm{old}}^{i}>\text{fitnes}s_{\mathrm{new}}^{i}\\ X^{i}(t+1)\;\;\text{else}\;\; \end{array}$$ (19)

Here, fitness values after and before update are denoted as $\text{fitnes}s_{\mathrm{old}}^{i}$ and $\text{fitnes}s_{\mathrm{new}}^{i}.$ This means that the search agents in the RBBMO algorithm do not remove earlier-found high-quality feature sets. This is vital in EV battery fault diagnosis, since the particular features are extremely valuable for predicting the battery concerns. The algorithm continues to explore novel solutions but also depends on the best solutions, making it converge on the optimal set of features. Thus, it enables the RBBMO to exploit the superior fault detection features while sustaining the capability to explore new possibilities. RBBMO efficiently explores the feature space for important defect indicators by imitating the natural foraging process. This ensures that only the most useful features are used to train the DL model. By lowering dimensionality and removing unnecessary or redundant features, it increases the model’s accuracy, resulting in quicker training and more accurate fault detection outcomes.

### 3.4 CAR-TATNet classifier

**Figure 3:**
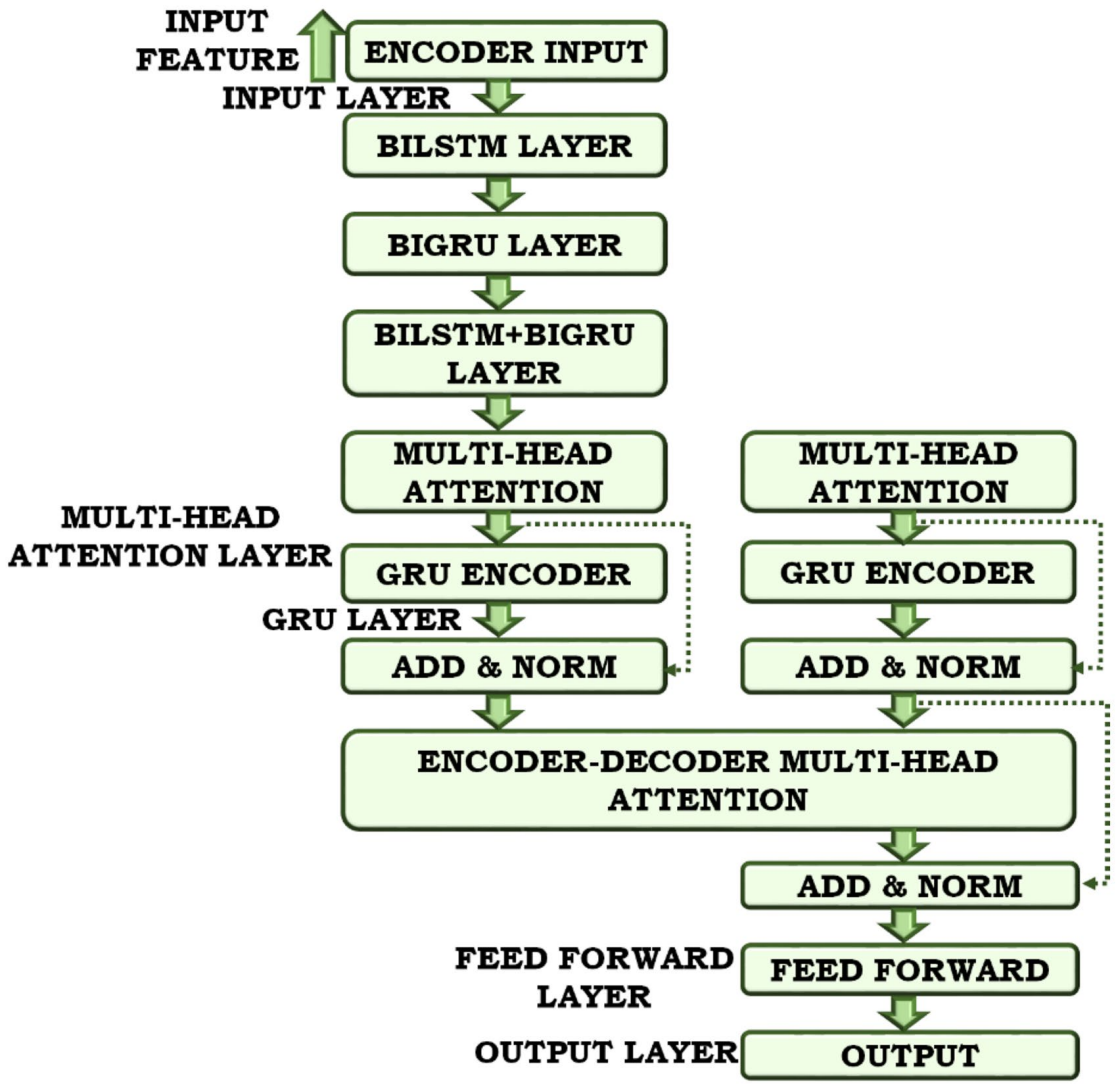
CAR-TATNet classifier’s structure.

CAR-TATNet is developed to improve the fault diagnosis of EV batteries by incorporating temporal dependencies and contextual awareness. It combines Context-Aware Recurrent Encoder (CA Encoder) based on GRU networks and Adaptive temporal transformer (ATT) that applies a multi-head mechanism for superior feature extraction and decision-making. It is appropriate for processing time series data from EV batteries. The structure of the CAR-TATNet classifier is illustrated in Figure 3. Table 1 presents the Hyperparameter settings of the CAR-TATNet classifier.

**Table 1:** Hyperparameter settings.

**Hyperparameter**	**Value**
Optimizer	Adam
Weight decay	1e-5
Learning rate	1e-4
Loss function	Categorical Cross entropy
Label smoothing	0.1
Batch size	32
Dropout rate	0.3
Epochs	20
GRU units	128
LSTM units	128
Transformer heads	8
Activation function	ReLU
Output activation	Softmax
Normalization	Layer normalization + batch normalization
Attention mechanism	Multi-head self attention

The CA Encoder functions as a dual-level GRU network. In the bottom level, the model processes the input time series data $x_{i}$ and updates the hidden state $\;h_{i}$ . It captures the historical dependencies in the battery’s function, which is computed as in Equation (20)

$${\hat{h}}_{i}=\mathrm{GR}U_{\text{lower}}({\vec{h}}_{i-1}^{a},E_{x_{i}})$$ (20)

Where the previous hidden state is ${\vec{h}}_{i-1}^{a}$ and input feature’s embedded representation is $E_{x_{i}}.$ The bottom-level GRU processes the past data’s sequence to summarize the battery’s operational history up to the present point. In the upper level, the model incorporates the hidden state from the bottom-level GRU with future context. This context-aware incorporation is managed by another GRU function that updates the hidden state as in Equation (21),

$${\vec{h}}_{i}^{a}=\mathrm{GR}U_{\text{higher}}({\hat{h}}_{i},{\vec{h}}_{i}^{c})$$ (21)

By enabling the model to encode past battery states and incorporate future predictions, the CA Encoder aids enhance the diagnostic power of the system by offering an understanding of the battery’s health over both past and predicted functional stages. The ATT improves CAR-TATNet by integrating multi-head attention to model long-term temporal dependencies between past battery data and future battery behavior. The input matrices pass via separate attention layers while the multi-head attention is applied to capture temporal relationships over distinct time frames. The attention mechanism is given in Equations (22–24),

$$V=XW^{V}$$ (22)

$$Q=XW^{Q}$$ (23)

$$K=XW^{K}$$ (24)

Here, the value, key, and query are denoted as V, K, and Q, respectively. The weight matrices that map the features to their associated value, key, and query representations are denoted as WV, WK, and W^Q, respectively The attention operation estimates the significance of each time step in relation to others as in Equation (25).

$$\text{Attention}\;(Q,K,V)=\text{Softmax}(\frac{QK^{T}}{\sqrt{d_{k}}})V$$ (25)

This operation allows the model to consider distinct time stages properly, concentrating on the most relevant data from both the future and past data for precise fault prediction. Subsequently, the output from multiple attention heads is concatenated and linearly converted to create the ending attention output. This is expressed in Equations (26–27).

$$\mathrm{hea}d_{i}=\text{Attention}(XW_{i}^{Q},XW_{i}^{K},XW_{i}^{V})$$ (26)

$$X_{\text{out}}=\text{Multi}-\text{head}(Q,K,V)=\text{Concat}(\mathrm{hea}d_{1},\cdots ,\mathrm{hea}d_{h})W^{o}$$ (27)

Where the output of the self-attention operation is denoted as $\mathrm{hea}d_{i},$ the transformation matrix applied to the concatenated attention heads are $W^{o}$ , and an output matrix is $X_{\text{out}}.$ A Gate recurrent unit (GRU) is exploited in CAR-TATNet to efficiently extract the temporal features from the EV battery data. To manage the flow of data, the GRU uses update and reset gates and updates the hidden state as given in Equations (28–31),

$$z_{t}=\sigma (W_{z}x_{t}+U_{z}h_{t-1}+b_{z})$$ (28)

$$r_{t}=\sigma (W_{r}x_{t}+U_{r}h_{t-1}+b_{r})$$ (29)

$${\hat{h}}_{t}=\tanh(W_{h}x_{t}+U_{h}(r_{t}⊙h_{t-1})+b_{h})$$ (30)

$$h_{t}=(1-z_{t})⊙h_{t-1}+z_{t}⊙{\hat{h}}_{t}$$ (31)

Where the update and reset gates are $\;r_{t}$ and $\;z_{t}$ and the candidate and past hidden states are ${\hat{h}}_{t}$ and $h_{t-1}$ , respectively. After that, the hidden features are passed via a feed-forward network for non-linear transformation, further improving the model’s capability to acquire composite patterns in the battery’s behavior as in Equation (32).

$$\mathrm{FFN}({\hat{h}}^{\mathrm{dec}})=\text{ReLU}({\hat{h}}^{\mathrm{dec}}W_{1}+b_{1})W_{2}+b_{2}$$ (32)

Where the learned weight matrices and biases are $\;W_{1},W_{2},b_{1},\text{and}\;b_{2}$ and output from the multi-head attention layer is ${\hat{h}}^{\mathrm{dec}}.$ By combining past battery data with predicted future states, the CAR-TATNet attains a context-aware representation of the battery’s behavior that is vital for appropriate and timely fault prediction in EV systems.

## 4 Result and discussions

This section presents the analyses and outcomes of the research conducted using a Python tool that validates the CAR-TATNet model to assess its effectiveness in diagnosing EV battery problems. According to experimental outcomes, the developed framework outperformed current state-of-the-art techniques, achieving better classification accuracy. The adaptive temporal transformer component improved detection reliability across a range of operating situations by effectively capturing dynamic fault changes and long-term dependencies.

The incorporation of contextual learning and optimization methods significantly reduced misclassification rates, as confirmed by a comparative study. Overall, the findings support the CAR-TATNet model’s robustness, computational effectiveness, and practicality for accurate and sensible EV battery problem diagnostics.

The battery operating parameters collected under both normal and faulty conditions for the dataset used in this study is sourced from https://www.kaggle.com/datasets/programmer3/ev-battery-and-drivetrain-fault-diagnosis. It comprises measurements taken throughout various cycles of charging and discharging, comprising current, temperature, voltage, State of Charge (SOC), and other battery management system (BMS) signals. The dataset allows for the analysis of fault characteristics and degradation patterns by capturing a variety of battery-fault scenarios.

The dataset details are listed in Table 2. The Data Type and Null Counts are depicted in Table 3, while Table 4 represents the computational complexity and training performance. During the training stage of the CAR-TATNet model, class weight balancing was added to address the dataset’s class imbalance problem. Minority classes were given higher weights, which improved classification fairness and generalization performance by permitting the model to focus more on under-represented fault categories The Software and Hardware Specifications are listed in Table 5.

**Table 2:** Dataset details.

Data shape	2,367 rows and 10 columns
Train and test split	80% TRAIN & 20% TEST
Column names	Time (ms), Voltage (V), Current (A), Temperature (°C), Motor speed (RPM), Hall code, estimated SOC (%), Ground truth SOC (%), Residual (%), Fault Label.
Input	Time (ms), Voltage (V), Current (A), Temperature (°C), Motor speed (RPM), Hall code, Estimated SOC (%), Ground Truth SOC (%), Residual (%).
Output	Fault label

**Table 3:** Data type and null counts.

Feature name	Data type	Missing values
Time (ms)	int64	0
Voltage (V)	float64	0
Current (A)	float64	0
Temperature (°C)	float64	0
Motor Speed (RPM)	int64	0
Hall Code	int64	0
Estimated SOC (%)	float64	0
Ground Truth SOC (%)	float64	0
Residual (%)	float64	0
Fault Label	Object	0

**Table 4:** Computational complexity and training performance.

**Parameter**	**Value**
Total epochs	20
Steps per epoch	60
Initial training time	21 s
Average epoch time	3–5 s
Final training accuracy	85.54%
Final validation accuracy	94.94%
Final validation loss	0.4214
Dl framework	TensorFlow/Keras

**Table 5:** Hardware and software specifications.

Component	Specification
Programming language	Python
Signal processing library	PyEMD
DL framework	TensorFlow/Keras
Operating system	Windows
GPU	NVIDIA GPU (if used)
RAM	16 GB
Development environment	Jupyter Notebook/Google Colab

**Figure 4:**
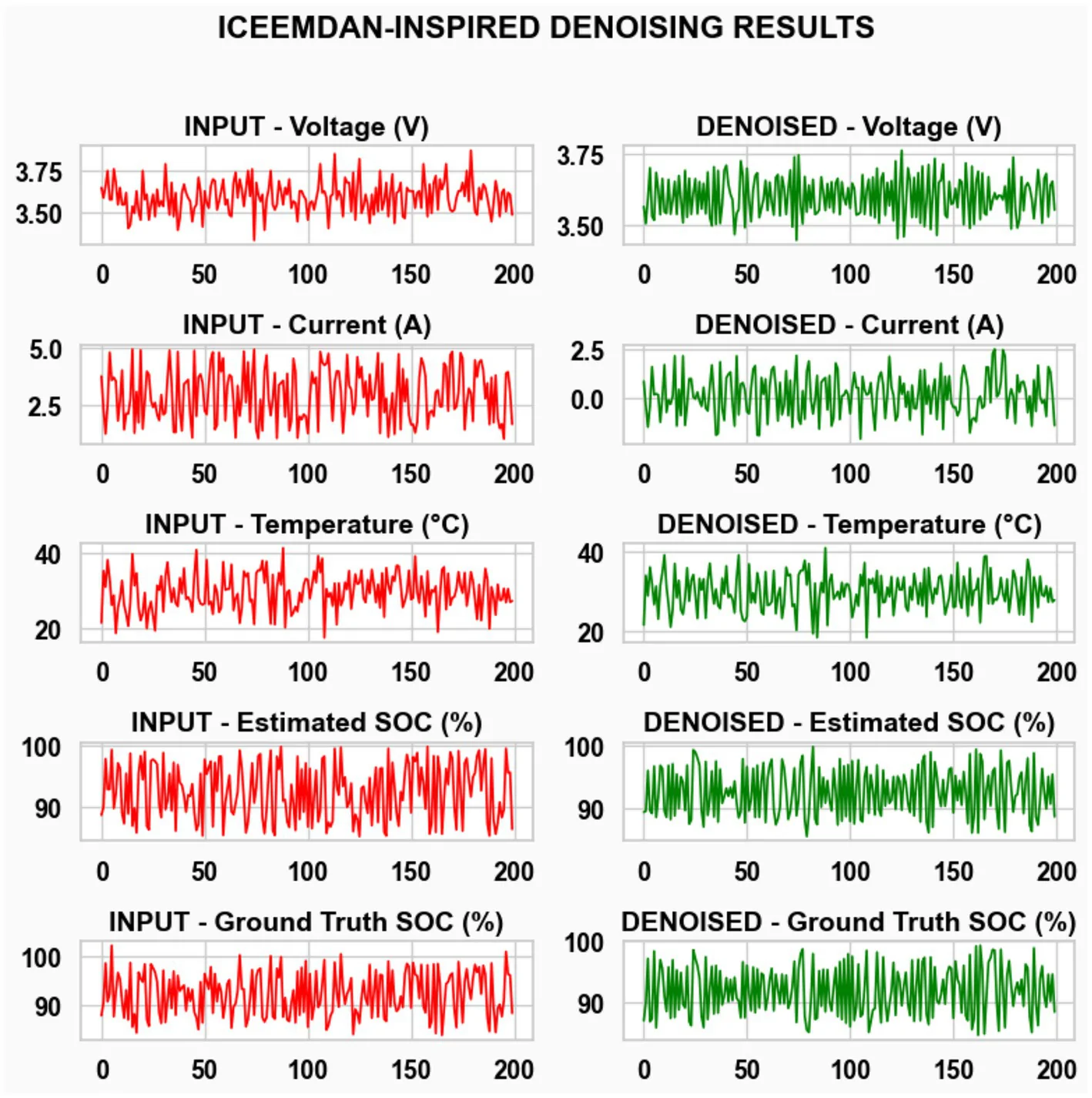
Denoising results.

The denoising results of different EV battery signals utilizing the ICEEMDAN technique are shown in the Figure 4. The original noisy input data for voltage, temperature, current, estimated SOC, and ground truth SOC are displayed in the left column and are all shown in red. The denoised signals are shown in green in the right column, demonstrating how well ICEEMDAN reduces noise while maintaining important battery parameters. The signal quality is much improved by this denoising process, which makes the features more appropriate for model training and defect identification. The denoising technique enhances model performance in EV battery failure detection by improving input signals.

**Figure 5:**
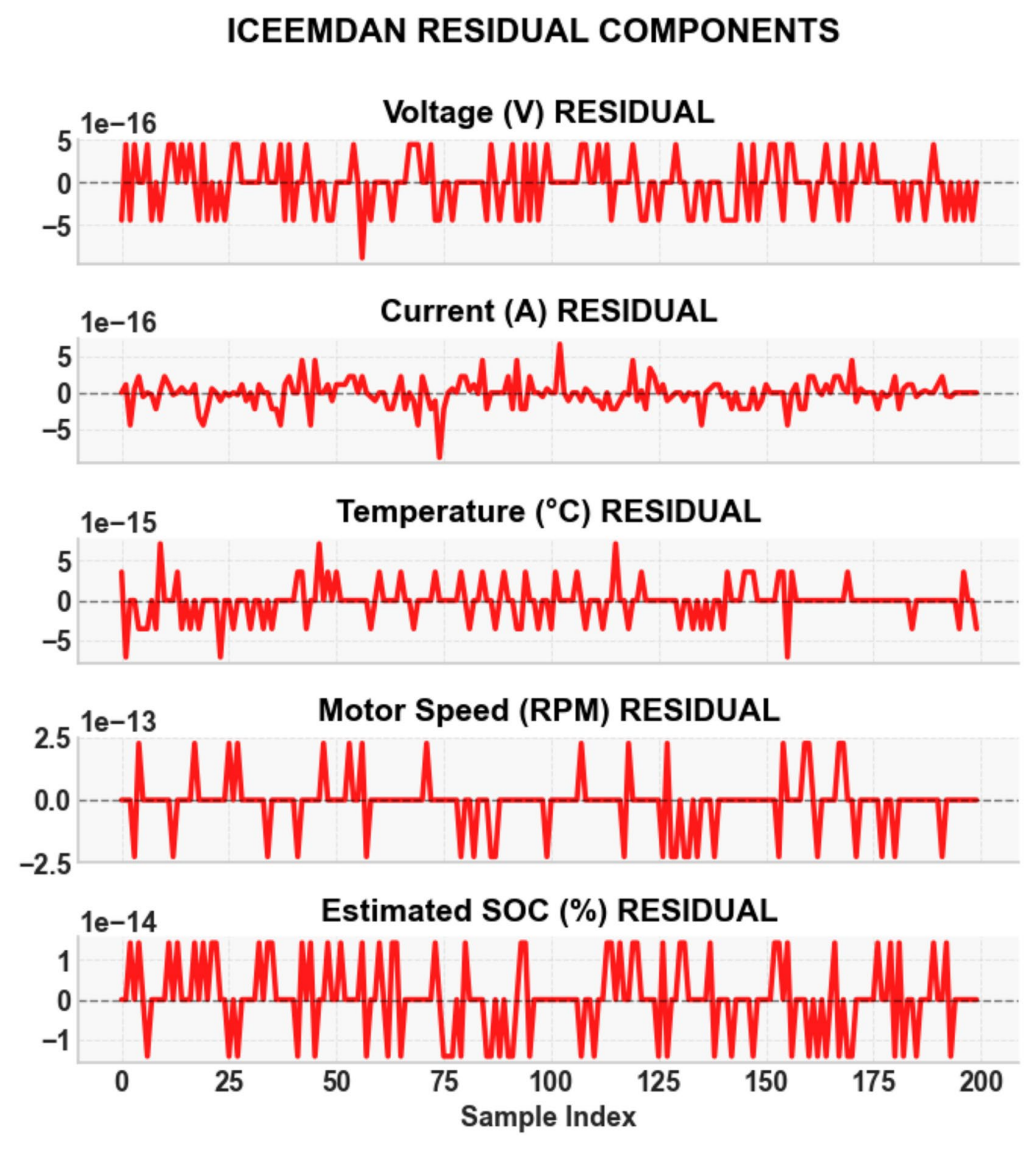
Residual components obtained after ICEEMDAN decomposition of voltage, current, temperature, motor speed, and estimated SOC signals.

The residual components, voltage, temperature, current, motor speed, and estimated SOC obtained by applying the ICEEMDAN to the EV battery dataset signals are depicted in the Figure 5. After the intrinsic mode functions and dominant signal features have been extracted, the remaining signal consists of noise and negligible fluctuations. The residual amplitudes being so tiny indicates that the decomposition process effectively caught the majority of the important information. The ability of ICEEMDAN to distinguish significant battery behavior from undesired noise and disturbances is demonstrated by the nearly negligible residual values across all parameters. This demonstrates how well the preprocessing phase enhances signal quality and supplies reliable input data for precise fault detection using the CAR-TATNet model.

**Figure 6:**
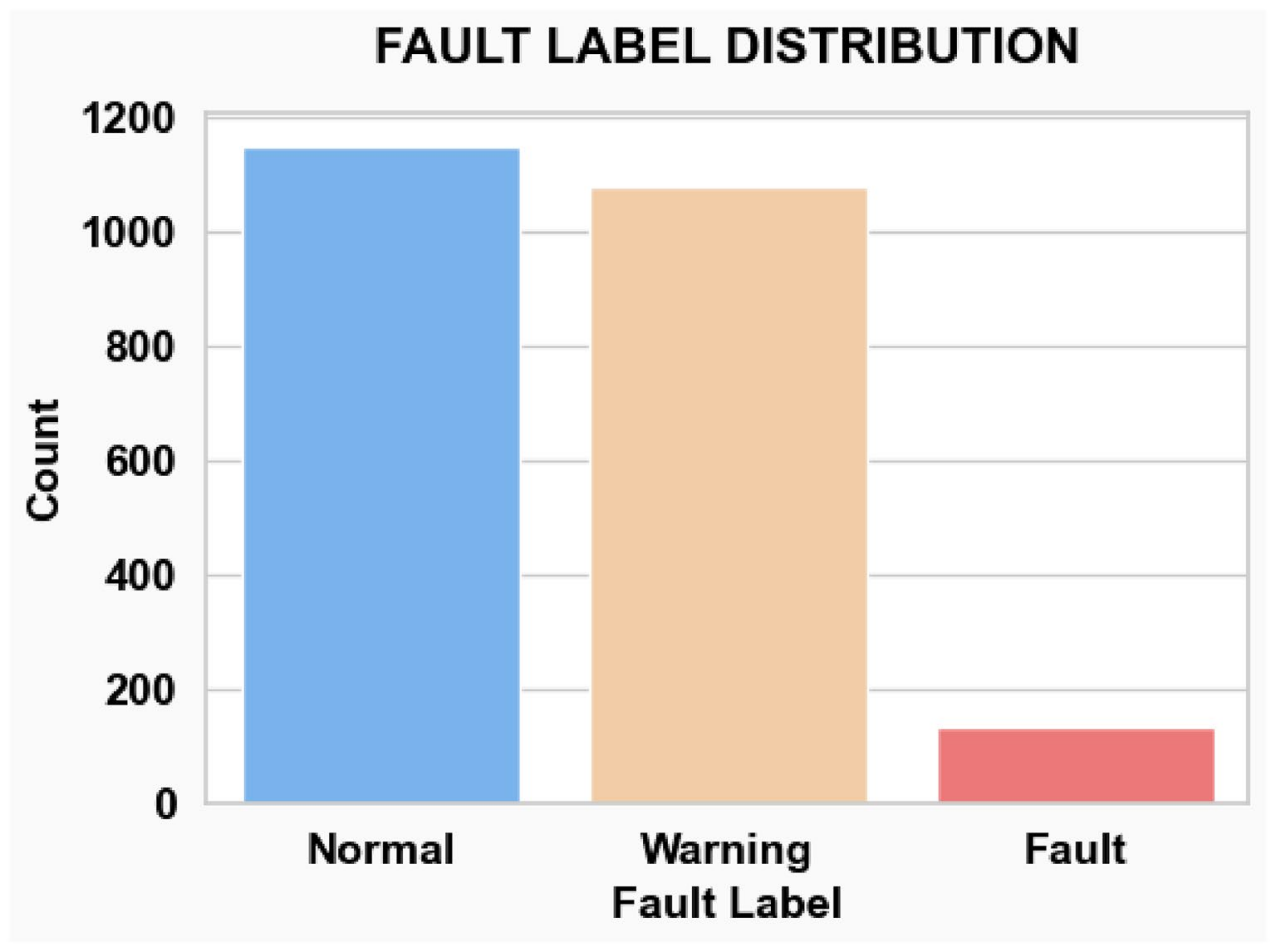
Distribution of fault labels.

The distribution of fault labels in the dataset from kaggle is used to diagnose battery problems in EVs, as displayed in Figure 6. The dataset has 2,367 rows and 10 columns. The graphic clearly illustrates the dataset’s imbalance, with the Normal label having the highest count, followed by the Warning label, and the Fault label having the lowest count. This distribution draws attention to a typical problem in fault detection jobs: the number of fault occurrences is outnumbered by normal conditions. In order to resolve the class imbalance, techniques like data augmentation or resampling are necessary. This imbalance might affect model training, potentially making it more difficult for algorithms to effectively detect uncommon fault events.

**Figure 7:**
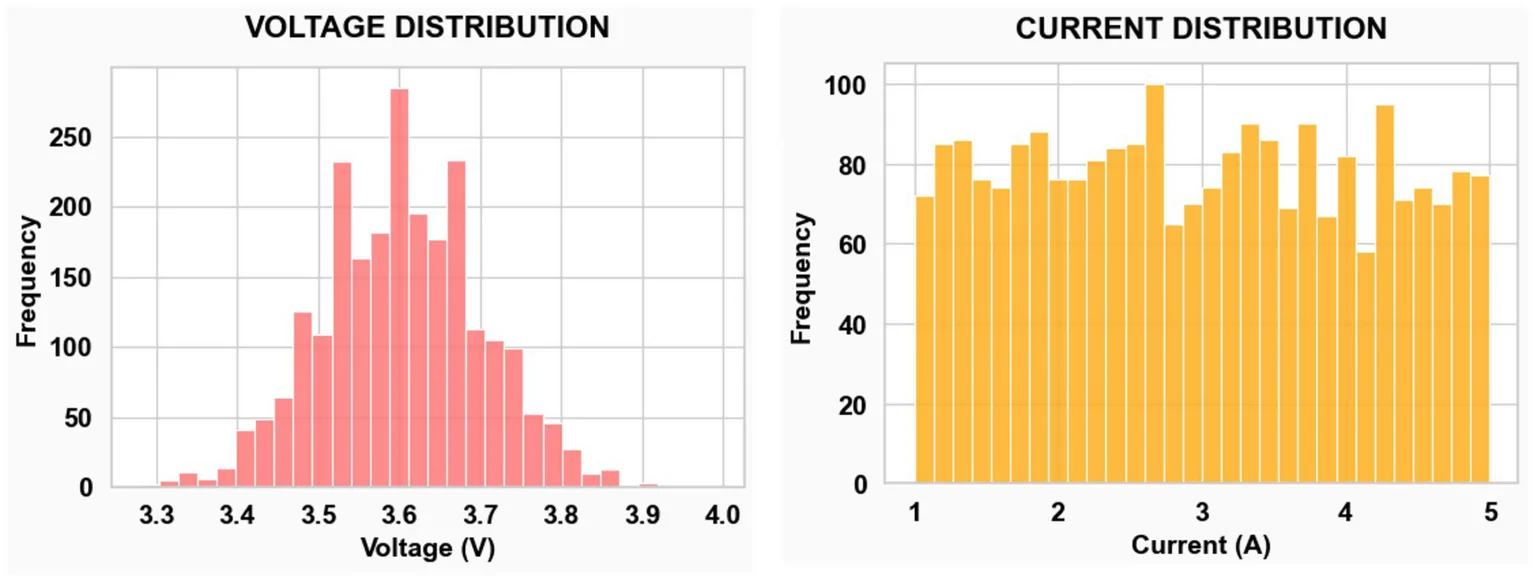
Distribution of current and voltage.

The distribution of two important EV battery properties is shown in Figure 7. The battery usually functions within a limited voltage range, as seen by the concentration of the voltage distribution around 3.6 V, a normal distribution, and fewer occurrences at the extremes (3.3 V and 4.0 V). The current distribution, on the other hand, is more uniform, with values ranging from 1A to 5A and no discernible concentration at any specific value, indicating that the battery operates under a range of load circumstances. By highlighting the battery’s typical operating characteristics, these distributions provide important information for fault diagnosis by pointing out departures from the predicted ranges.

**Figure 8:**
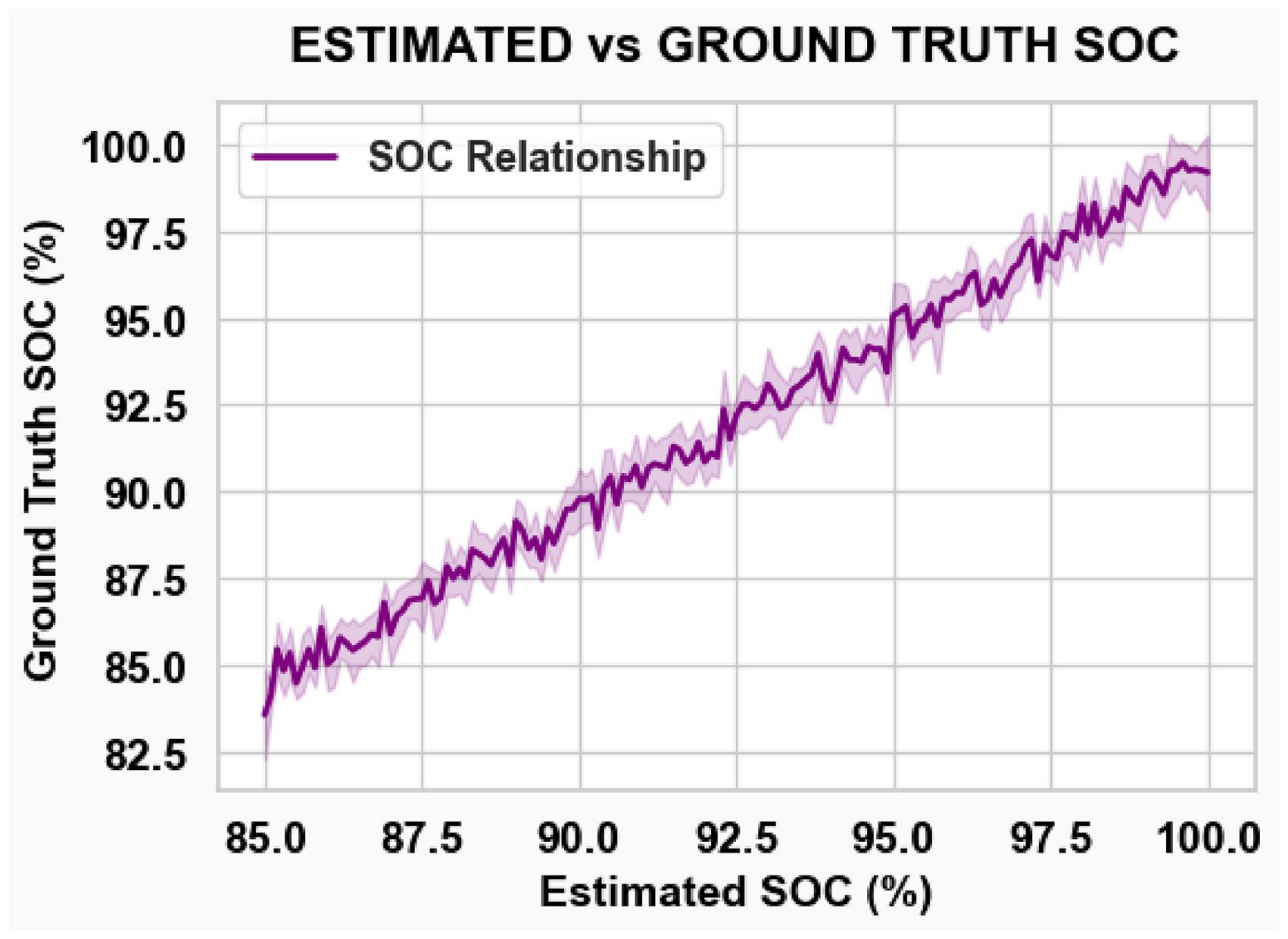
SoC relationship.

The link between an EV battery’s Estimated SOC and Ground Truth SOC is depicted in Figure 8. It shows a link between the Ground Truth SOC on the y-axis and the Estimated SOC on the x-axis. The Ground Truth SOC climbs in tandem with the Estimated SOC, signifying that the estimated values roughly correspond to the real battery charge. The estimation’s variability or uncertainty is shown by the shaded region surrounding the line. Overall, the two SOC values near alignment indicates that the estimation model is reasonably accurate, which is important for efficient battery management and defect detection in EVs.

**Figure 9:**
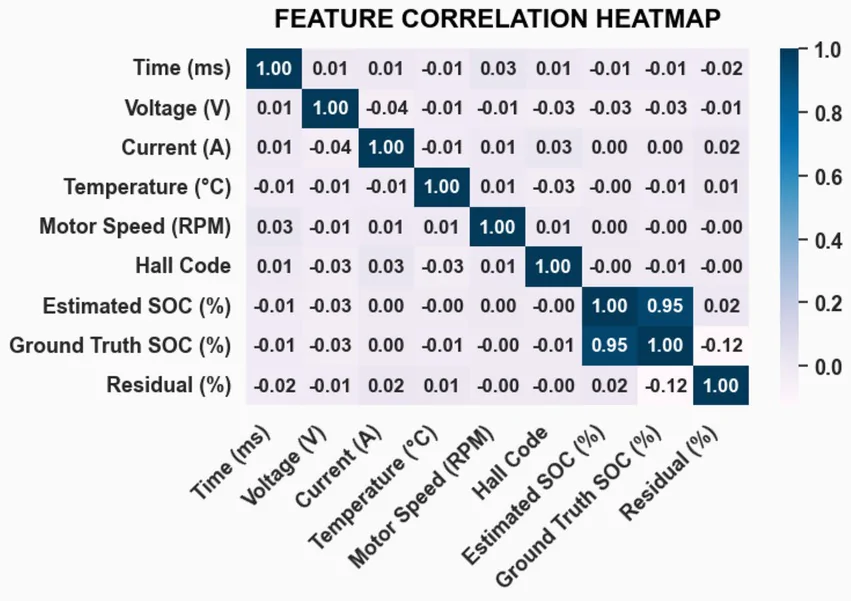
Feature correlation heat map.

A feature correlation heat map showing the connections between different EV battery dataset features is shown in Figure 9. The heat map, whose values range from −1 to 1, shows how strongly each attribute corresponds with the others. The estimated SOC and the actual SOC closely match, as evidenced by the strong positive correlations between the two. Furthermore, there is a moderately negative association between Residual and Estimated SOC and Ground Truth SOC, indicating a modest decrease in residual error as SOC rises. There are only modest relationships between the SOC values and the other characteristics, such as voltage, current, and temperature. It is useful for choosing features and figuring out which parameters are most important for problem detection and battery-state monitoring.

**Figure 10:**
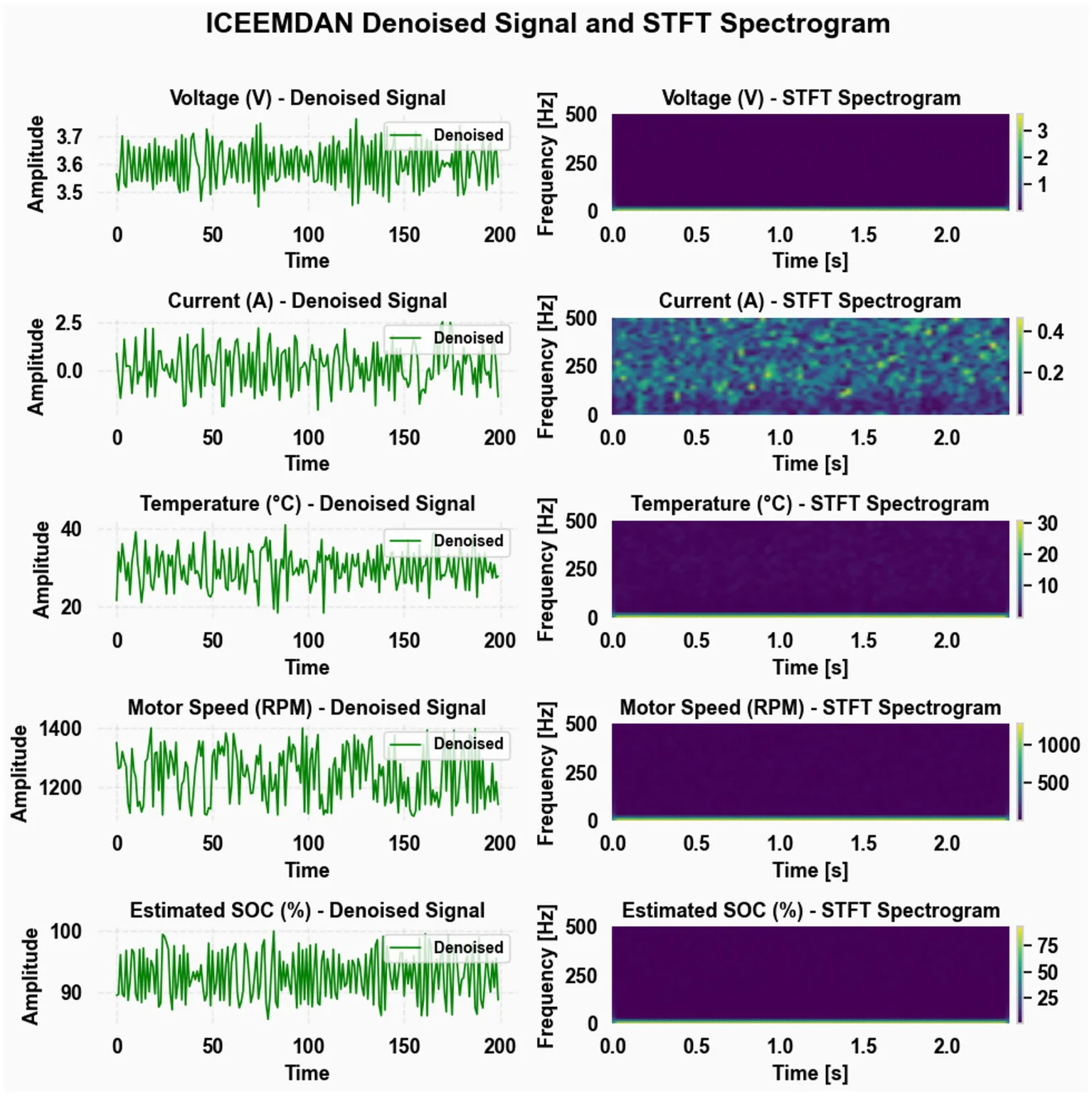
Denoising and time-frequency analysis.

The denoising and time-frequency analysis of various important EV battery metrics using ICEEMDAN and STFT are depicted in Figure 10. The denoised voltage, current, temperature, motor speed, and estimated SOC data are shown on the left. The revised data following noise reduction is represented by these denoised signals, which are shown in green. The accompanying STFT spectrograms on the right display each parameter’s frequency content with time. Denoising and time-frequency analysis work together to improve signal quality and offer insightful information about the dynamic behavior of EV battery parameters, which helps with performance monitoring and issue identification.

**Figure 11:**
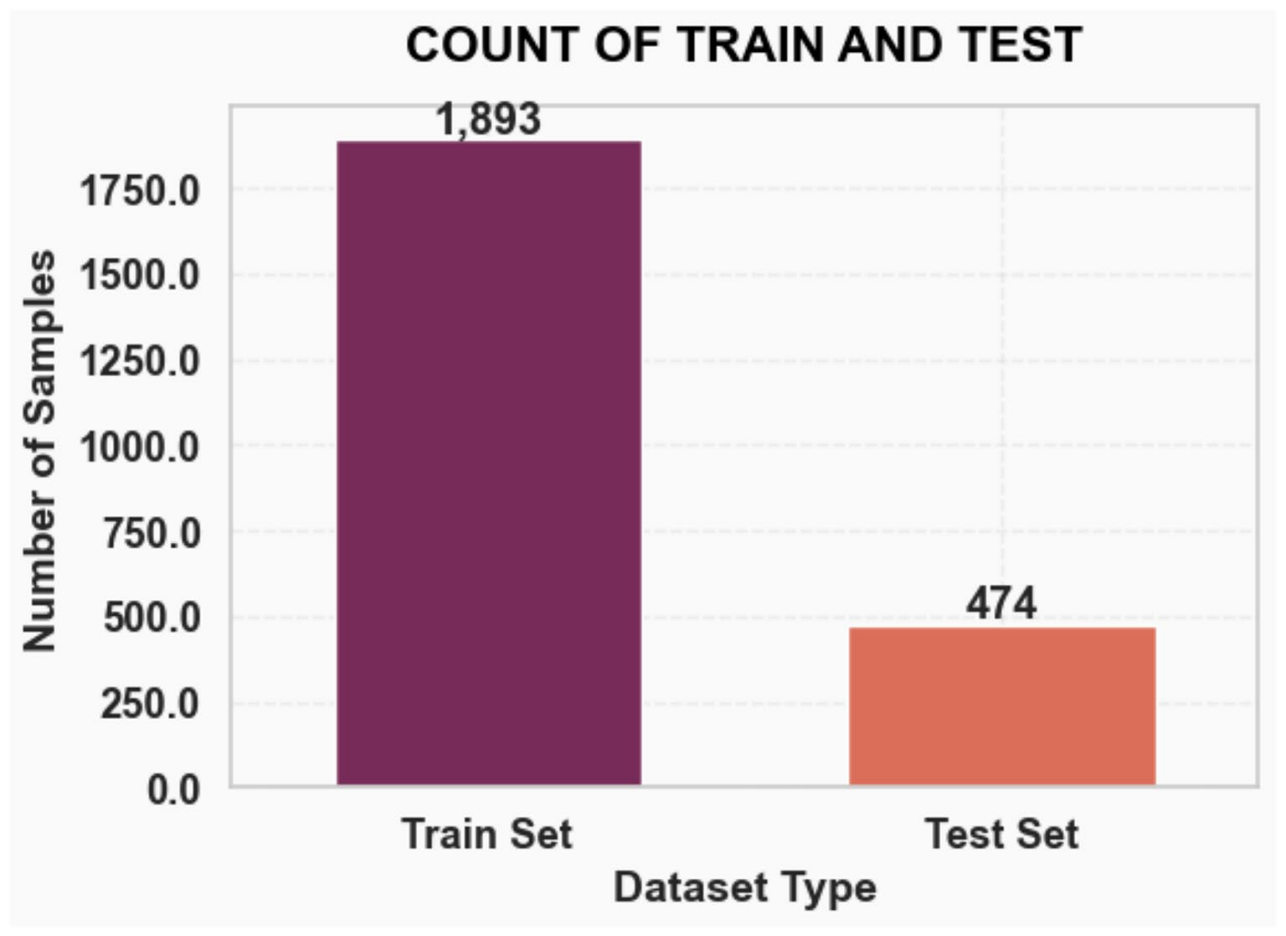
Distribution of samples.

The distribution of samples between the dataset’s training and test sets is depicted in Figure 11. The training set has 1,893 samples, which is more than the test set’s 474 samples, according to the bar chart. This sample size imbalance reflects a common practice in DL, where a smaller portion of data is set aside for testing to assess model performance, while the bulk is used for training to ensure the model learns properly. While the test set enables the evaluation of the model’s capacity to estimate new, unobserved data, the big training set improves the model’s generalization.

**Figure 12:**
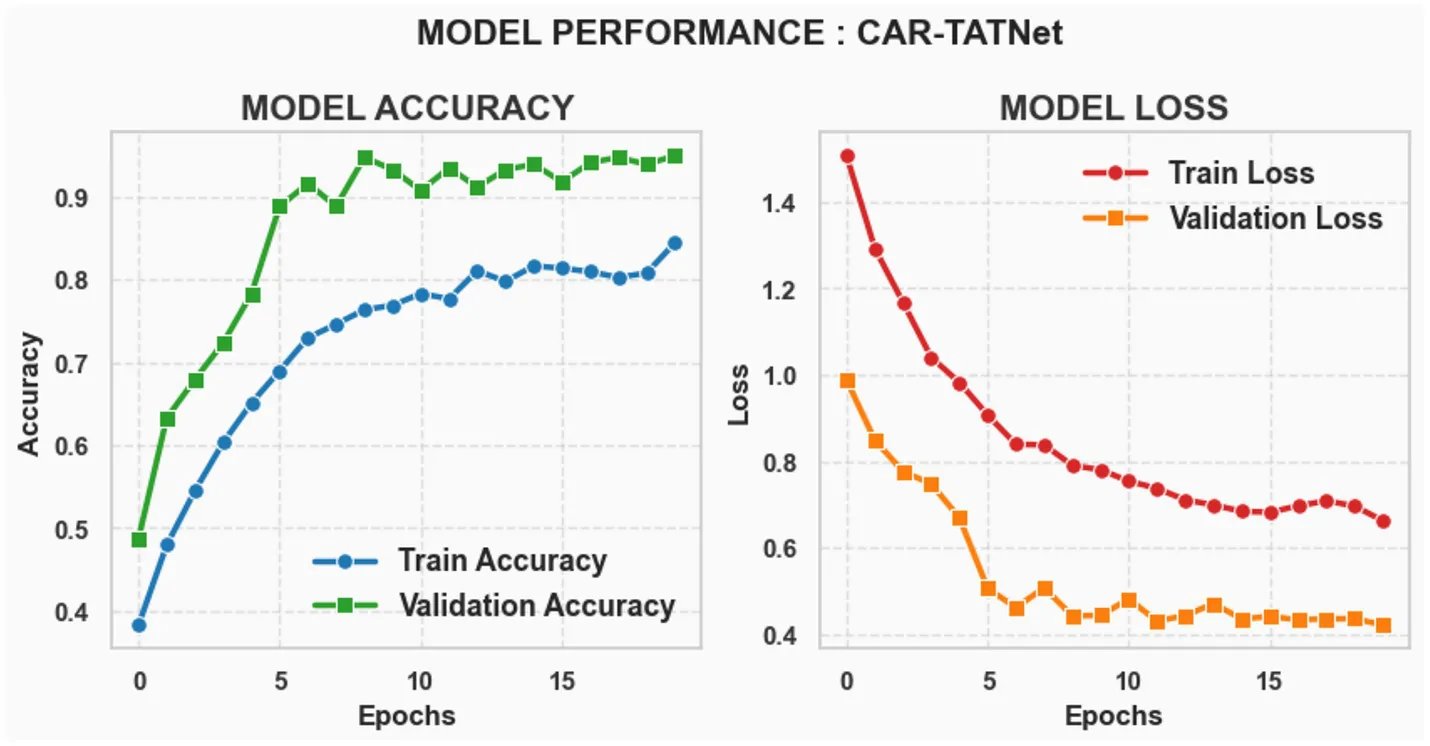
Accuracy and loss curves of the CAR-TATNet.

The accuracy and loss curves of CAR-TATNet model’s performance measures over training epochs are exhibited in Figure 12. The model accuracy graph on the left displays the accuracy of training and validation across epochs. The training accuracy climbs dramatically and stabilizes as training goes on, while the validation accuracy also rises but levels off slightly after reaching a certain point, suggesting that the model is generalizing well to new data. The model’s decreasing error rate is demonstrated by the training loss’s fast decline and the validation loss’s slower decline. The stabilization of both accuracy and loss values suggests that the CAR-TATNet model is successfully learning the underlying patterns in the data and is not overfitting.

**Figure 13:**
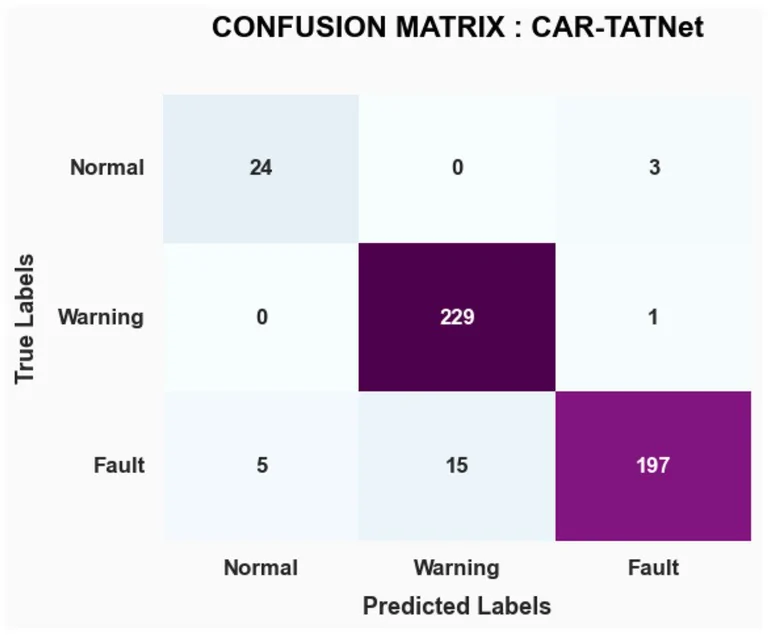
Confusion matrix.

The CAR-TATNet model’s effectiveness in identifying the three fault labels is displayed as the confusion matrix in Figure 13. With 229 out of 230 samples properly identified, the Warning class performed the best, demonstrating outstanding early failure state detection. Particularly, the model’s capacity to distinguish between healthy and deteriorated states was confirmed by the fact that no Warning samples were incorrectly classified as Normal. The majority of fault samples (197) were accurately detected, although a small percentage were forecasted as warning, representing that severe faults have similar characteristics.

**Figure 14:**
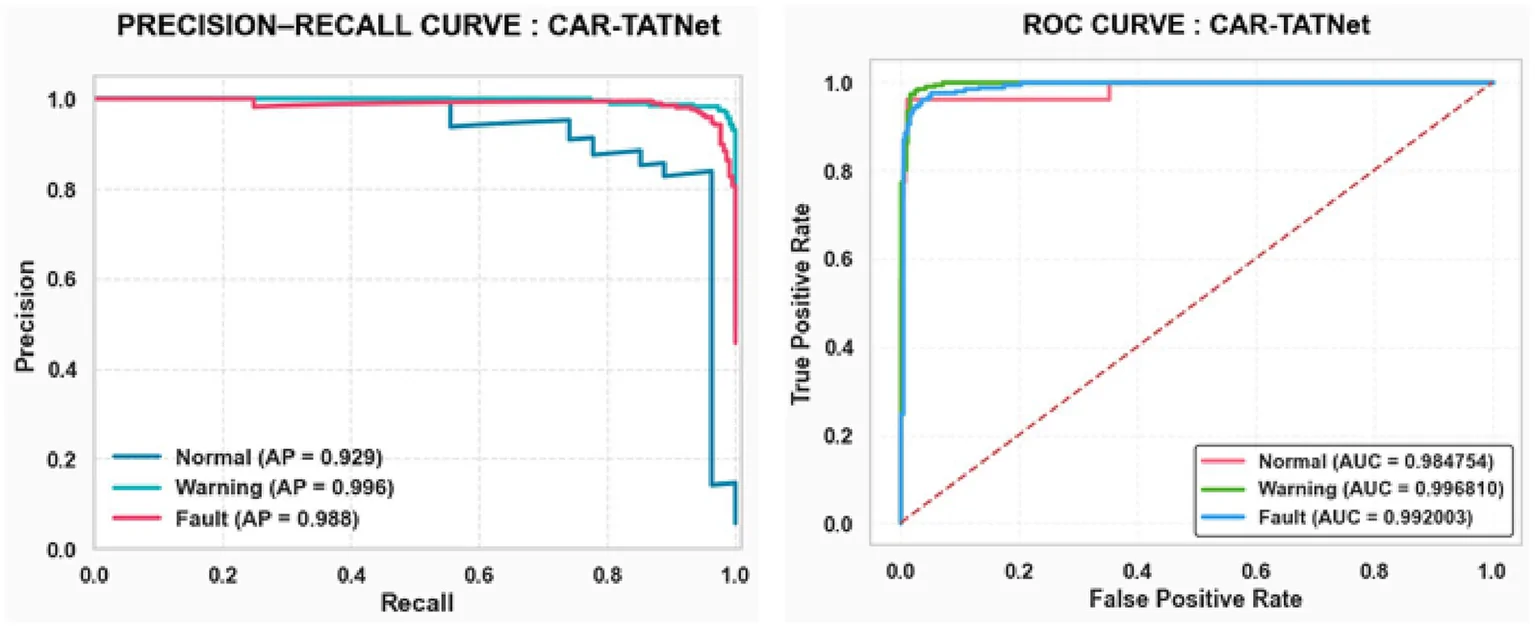
Precision-recall and ROC curve.

Two evaluation measures for the CAR-TATNet model are shown in Figure 14. The model performs exceptionally well across all classes in the Precision-Recall Curve, with the Warning class having the highest Average Precision (0.996), followed by the Fault class (0.988) and the Normal class (0.929). It suggests that, while the Normal class performs somewhat worse, the model is especially good at recognizing the Warning and Fault categories. With high Area Under the Curve values for each class, Normal (0.9847), Warning (0.9968), and Fault (0.9920), the ROC Curve shows that the model is better able to differentiate between all classes. The precision and ROC curve measurements show that the CAR-TATNet model works well, especially when it comes to identifying Warning and Fault categories.

**Figure 15:**
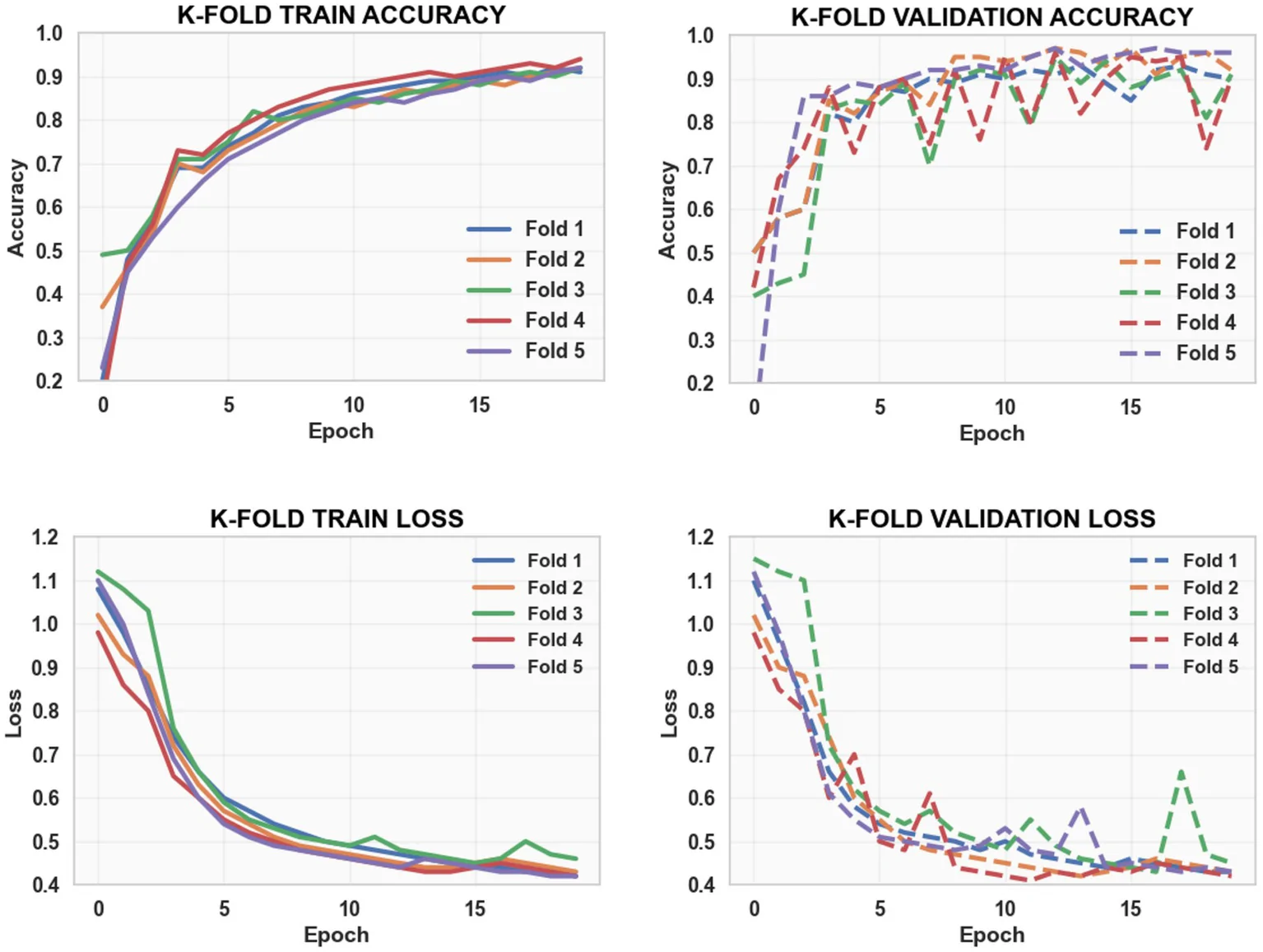
Training and validation using 5-fold cross-validation.

**Figure 16:**
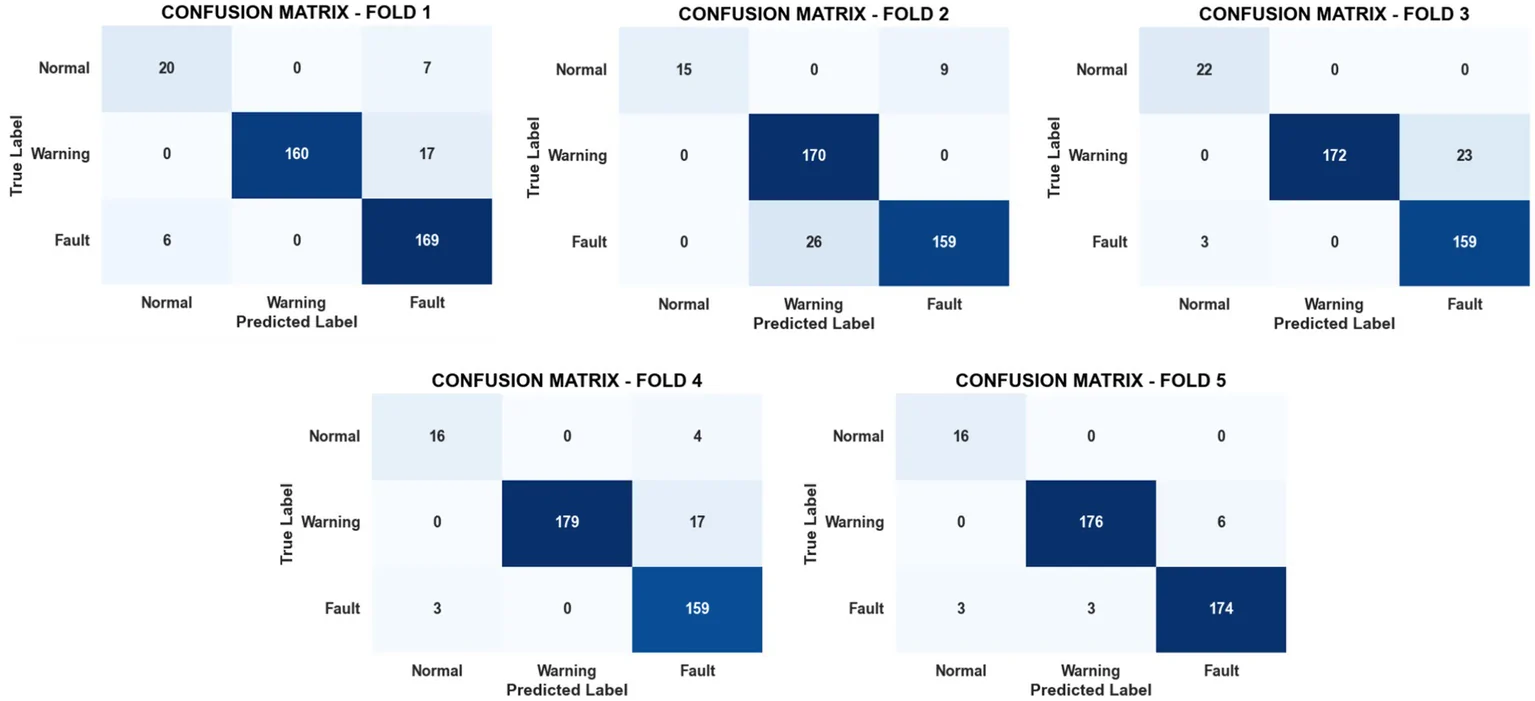
Confusion matrices.

The robustness, stability and generalization capabilities of the CAR-TATNet model are evaluated using 5-fold cross-validation. By testing the model across several data partitions and lowering the possibility of performance bias brought on by a single data split, the addition of k-fold cross-validation offers a more reliable statistical evaluation. The resilience of the CAR-TATNet model for EV battery issue diagnosis is shown by the 5-fold cross-validation, as seen in Figure 15. Stable learning and effective feature extraction are demonstrated by the training accuracy, which converged above 90% throughout all five folds, while the training loss continuously dropped. In a similar vein, throughout the training phase, the validation accuracy stayed high, averaging between 90 and 97% in most folds with low validation loss values. Thus, the CAR-TATNet is an accurate framework for intelligent EV battery defect detection and classification under various data distributions Figure 16.

The 5-fold cross-validation confusion matrices for classification of EV battery faults is presented in Figure 16. With a large number of properly classified samples centered along the diagonal of each confusion matrix, the model differentiated between the three battery health states across all folds. The model’s efficacy in identifying battery anomalies and critical fault states is demonstrated by the exceptionally high detection rates attained by the Warning and Fault classes. Consistent performance over all five folds validates the resilience and generalization capabilities of CAR-TATNet for precise EV battery condition monitoring and fault detection, as shown in Figure 16.

**Figure 17:**
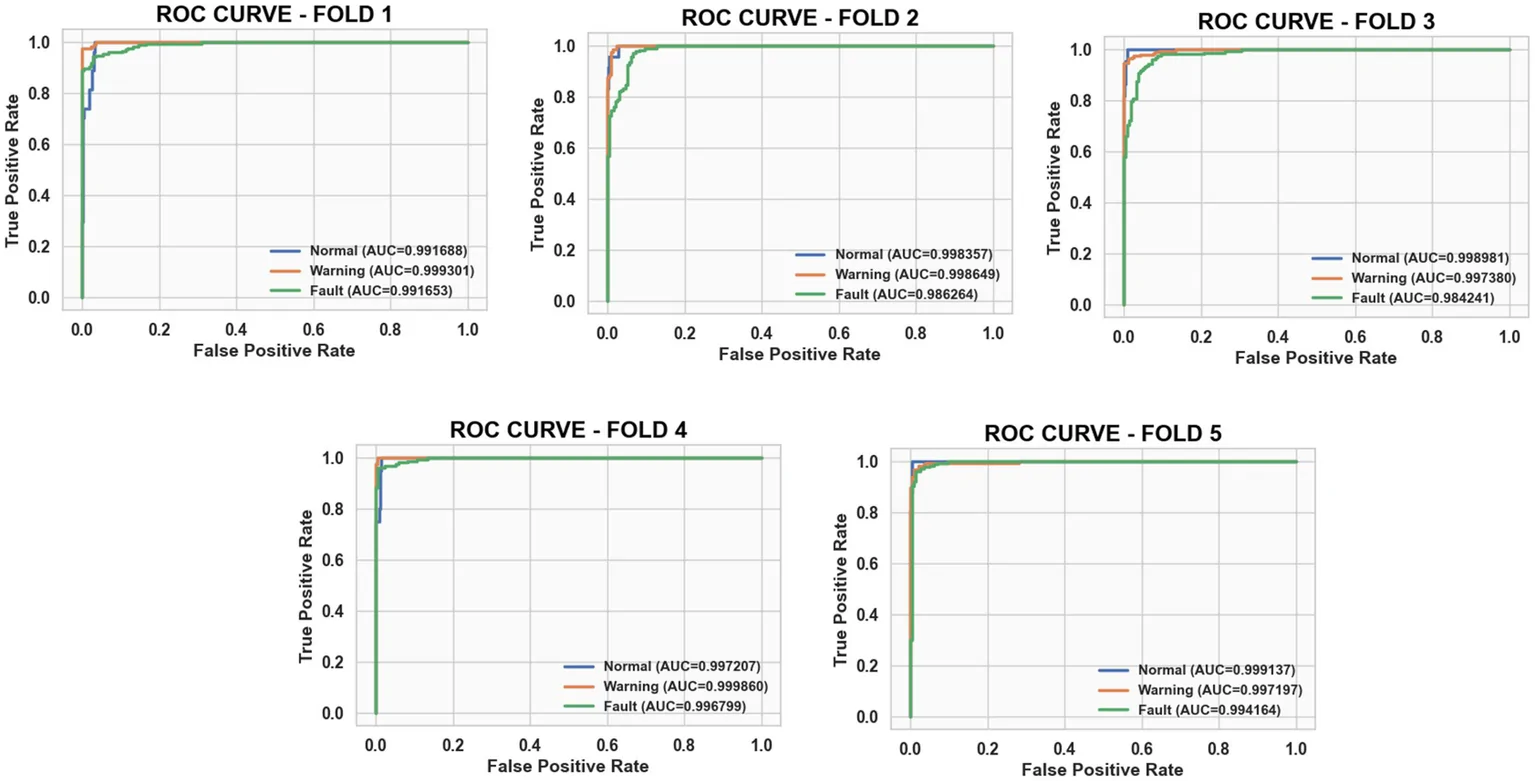
ROC curves.

The exceptional classification capabilities of the CAR-TATNet model for EV battery defect diagnostics are shown by the ROC curves from the 5-fold cross-validation, as represented in Figure 17. The ROC curves for the Normal, Warning, and Fault classes are clustered in the upper-left corner across all folds, suggesting a low false-positive rate and a high true-positive rate. The model’s unique capability to distinguish between various battery health situations is confirmed by the AUC values, which are constantly near 1.0. The CAR-TATNet offers extremely accurate and dependable fault detection performance, which makes it ideal for EV battery diagnostic applications.

**Figure 18:**
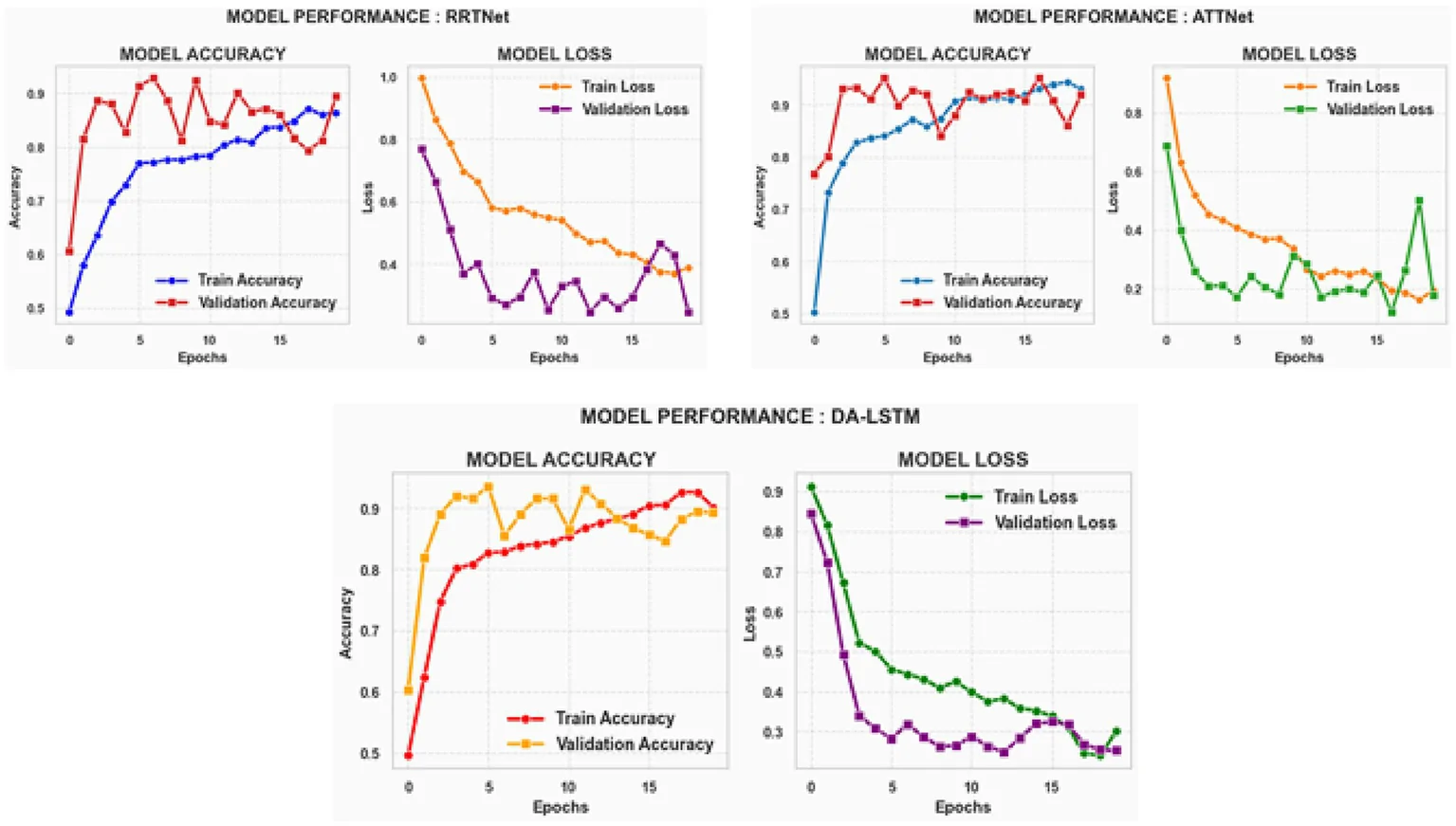
Accuracy and loss curves.

The accuracy and loss curves of RRTNet, ATTNNet, and DA-LSTM performance over training epochs are shown in Figure 18. When it comes to RRTNet, the training accuracy increases gradually but the validation accuracy varies, suggesting some instability in generalization. Additionally, the validation loss shows instability while the training loss dramatically declines. ATTNNet performs well, with both training and validation accuracy increasing rapidly and stabilizing and both training and validation loss curves improving steadily without exhibiting overfitting. Additionally, DA-LSTM exhibits good model performance without overfitting, with a sharp increase in accuracy and a consistent decay in training and validation loss. While RRTNet exhibits some instability during the validation phase, indicating potential for improvement in generalization, ATTNNet and DA-LSTM demonstrate consistent and robust outcomes.

**Figure 19:**
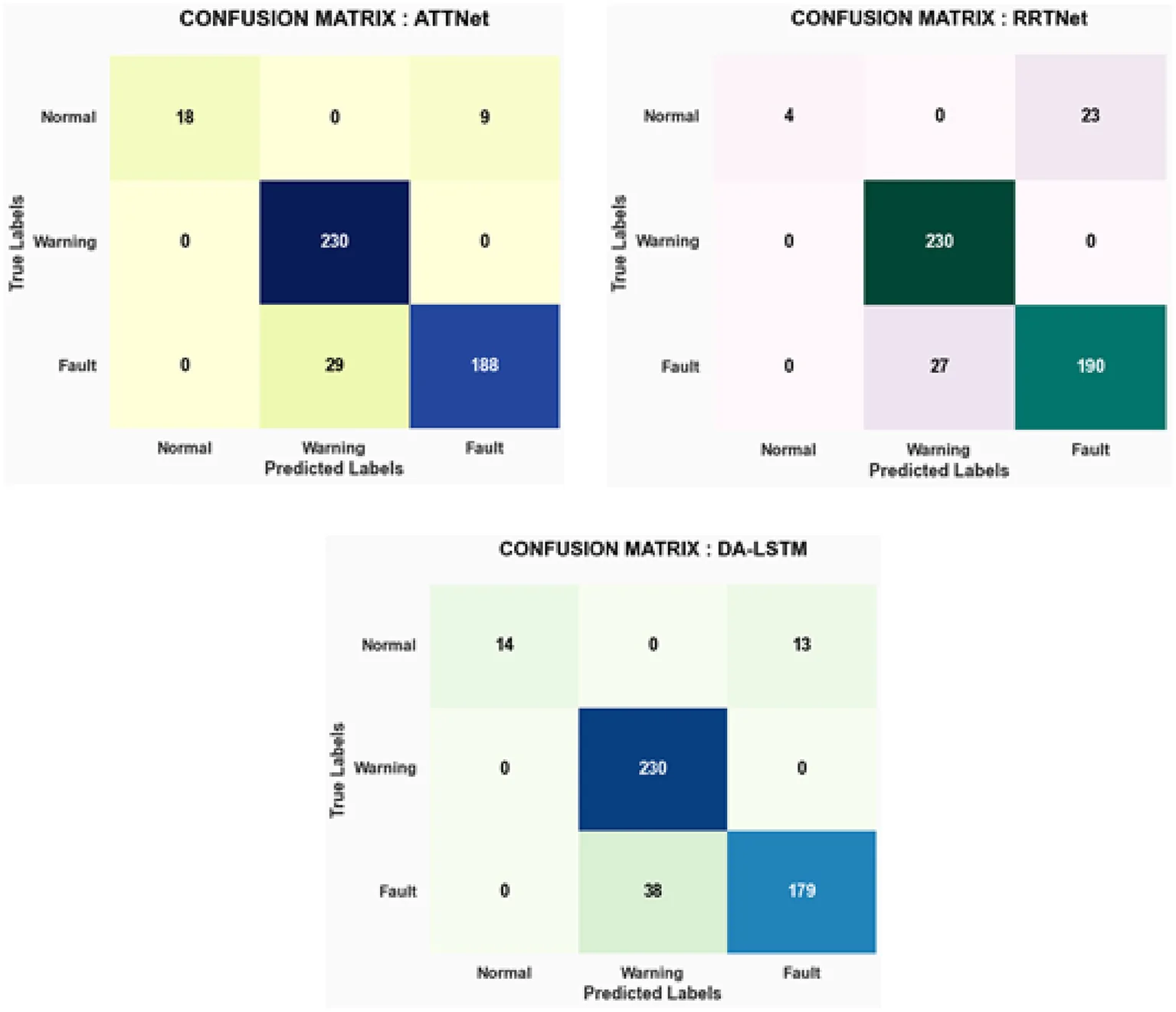
Confusion matrices.

Confusion matrices for three distinct models, namely RRTNet, ATTNNet, and DA-LSTM, are displayed in Figure 19. The model correctly predicted 230 Warning cases in the RRTNet matrix but incorrectly classified 4 Normal instances as Fault and 27 Fault instances as Warning. With 230 accurate Warning predictions, the ATTNNet model performs well, and it misclassified 18 Normal cases and 9 Fault. Similarly, 230 Warning cases were properly predicted by the DA-LSTM model, with minor misclassifications between Normal and Fault. With minor misclassifications mostly between the Normal and Fault categories, all three models performed well overall, demonstrating their efficacy in identifying the different kinds of faults.

**Figure 20:**
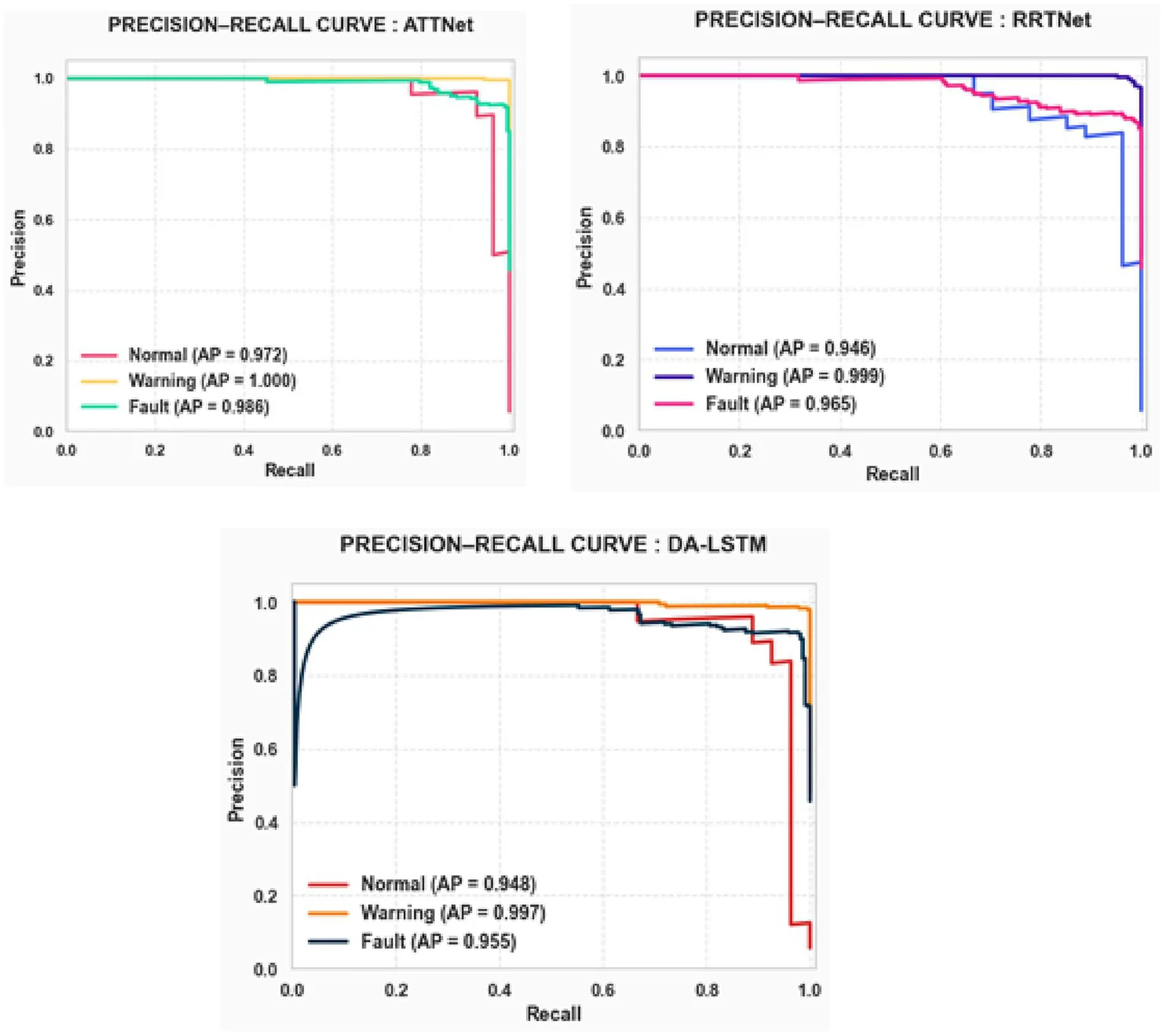
Precision-recall curves.

The Precision-Recall curves for the RRTNet, ATTNNet, and DA-LSTM models are displayed in Figure 20. The model performs well in RRTNet, with the Warning class having the greatest Average Precision (AP) of 0.999, followed by Fault (0.965) and Normal (0.946). While Fault and Normal exhibit a reasonable mix between precision and recall, the Warning class curve displays almost perfect precision. With a flawless AP of 1.000 for Warning and high AP values of 0.972 for Normal and 0.986 for Fault, ATTNNet exhibits exceptional performance. The fault and warning curves both exhibit high recall and precision. With an AP of 0.997 for Warning, 0.955 for Fault, and 0.948 for Normal, DA-LSTM likewise performs well, exhibiting excellent precision in all categories, but especially for Warning.

**Figure 21:**
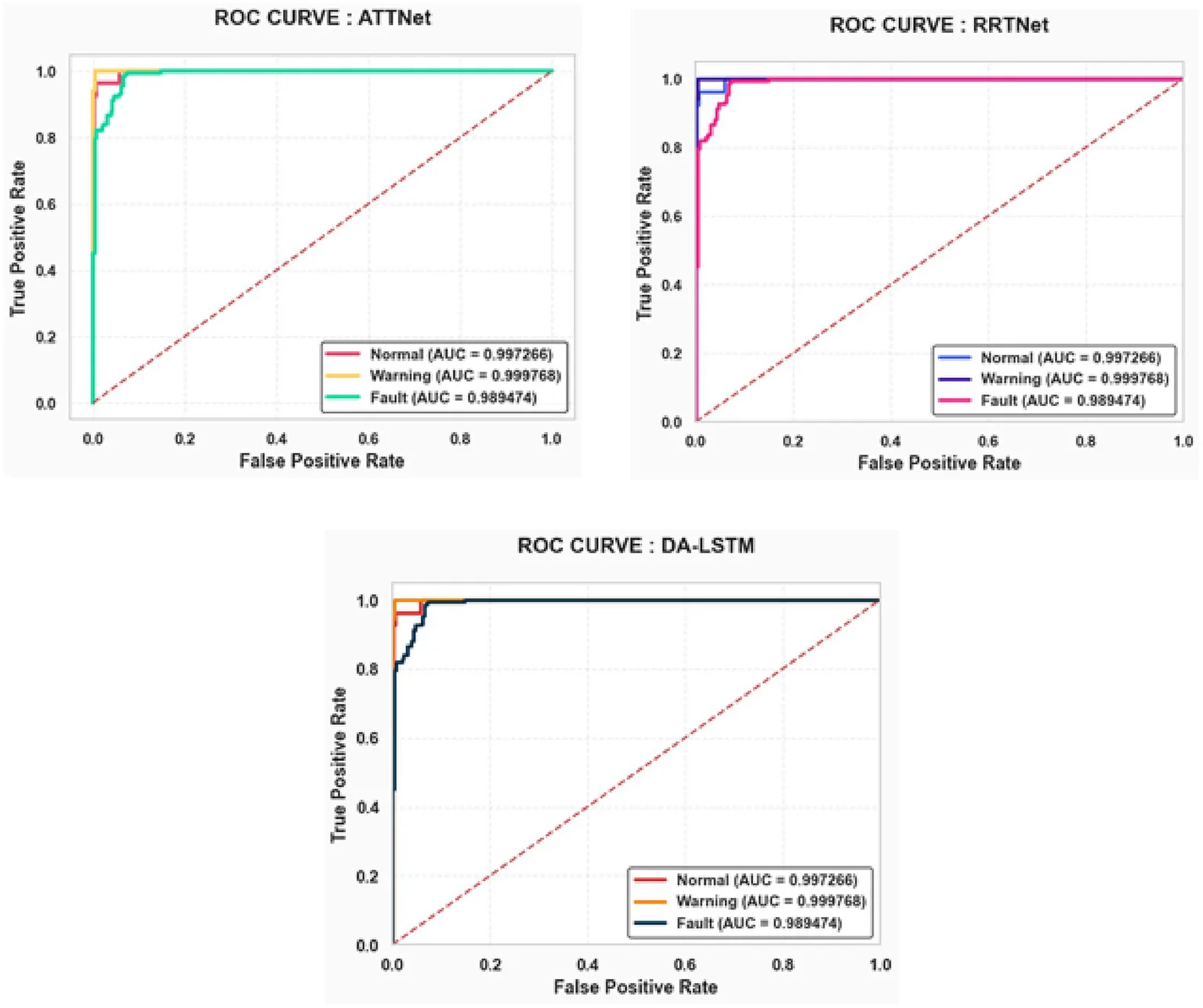
ROC curves.

The ROC curves for the ATTNNet, RRTNet, and DA-LSTM models are displayed in Figure 21, demonstrating their capacity to differentiate between the Normal, Warning, and Fault categories. The model successfully distinguishes between the categories in ATTNNet, as evidenced by the AUC values of 0.997766 for Normal, 0.997478 for Warning, and 0.994874 for Fault. With AUC values of 0.997766 for Normal, 0.997976 for Warning and 0.994747 for Fault, RRTNet likewise performs well. Similarly, DA-LSTM performs well in all categories, achieving AUC values of 0.997766 for Normal, 0.997476 for Warning, and 0.994874 for Fault. The model demonstrates superior classification performance and the capacity to successfully differentiate between various fault types.

**Figure 22:**
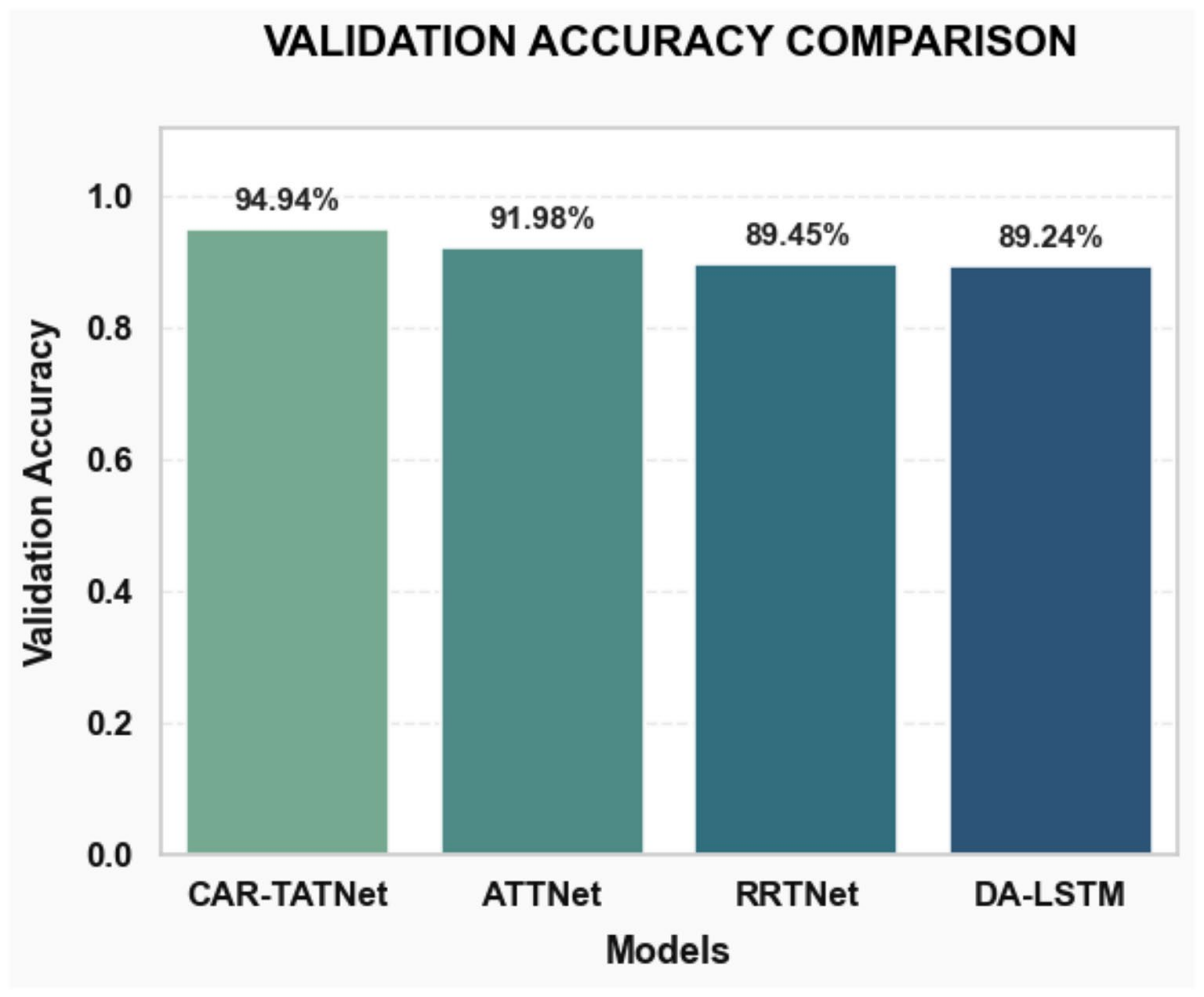
Validation accuracy.

The validation accuracy of four models, namely CAR-TATNet, ATTNNet, RRTNet, and DA-LSTM, is compared in Figure 22. With a validation accuracy of 94.94%, CAR-TATNet performs better than the rest, followed by ATTNNet with 91.98%. With validation accuracies of 89.45 and 89.24%, respectively, RRTNet and DA-LSTM perform marginally worse. This comparison shows that CAR-TATNet performs better than ATTNNet, whereas RRTNet and DA-LSTM provide comparable validation accuracy, although at a lesser level than the two models.

Table 6 depicts the ablation study illustrating the overall performance. The greatest performance reduction occurred when the CAR-TATNet design was removed, where accuracy decreased from 94.94 to 88.63%, highlighting its vital role in fault classification. The accuracy decreased to 92.76, 90.95, and 91.87%, respectively, when RBBMO, STFT, and ICEEMDAN were removed, demonstrating that each module aids in feature extraction and representation learning. With 94.94% accuracy, 91.54% precision, and 92.20% F1-score, the developed model demonstrated the best performance and superior fault identification ability. Furthermore, the model’s ability to accurately identify between fault states while reducing false alarms is demonstrated by the 97.06% specificity.

**Table 6:** Ablation study.

Model	Accuracy	Precision	Recall	F1-score	Specificity
Without CAR-TATNet	88.63	84.95	86.71	85.82	91.766
Without RBBMO	92.76	89.94	90.65	90.24	95.41
Without STFT	90.95	87.31	88.54	87.92	93.84
Without ICEEMDAN	91.87	88.42	89.76	89.08	94.63
Proposed model	94.94	91.54	93.08	92.2	97.06

With the greatest specificity of 0.9706, the CAR-TATNet model has better diagnostic performance than DA-LSTM, ATTNNet, and RRTNet models, demonstrating its remarkable ability to correctly identify normal battery conditions with few false alarms. When compared to alternative methods, it achieves a recall of 0.9308, demonstrating increased sensitivity in identifying real battery problems, as seen in Table 7. A steady and well-balanced fault classification performance is shown by the F1-score of 0.9220. The model guarantees dependable and precise fault forecasts with a precision of 0. 9,154. Thus, the obtained accuracy of 0.9494 demonstrates that CAR-TATNet performs better than other models in managing intricate and changing EV battery failure diagnosis scenarios.

**Table 7:** Analysis of performance metrics.

Classifiers	Specificity	F1-score	Recall	Precision	Accuracy
DA-LSTM	0.9312	0.8273	0.7811	0.9302	0.8924
ATTNet	0.9487	0.8830	0.8443	0.9474	0.9198
RRTNet	0.9333	0.6954	0.6746	0.9290	0.8945
CAR-TATNet	0.9706	0.9220	0.9308	0.9154	0.9494

The AUC-ROC and Jaccard Index comparison outcomes demonstrate the CAR-TATNet model’s effectiveness, as revealed in Table 8. The model’s outstanding ability to discriminate between defective and normal battery states across a range of threshold settings is demonstrated by its remarkable AUC-ROC value of 0.9912. With the greatest Jaccard Index of 0.8587 in terms of similarity measurement, CAR-TATNet outperforms other models in terms of the overlap between predicted and actual fault classes. The Jaccard Index indicates that the suggested model offers superior diagnosis consistency, despite ATTNet’s marginally higher AUC-ROC. In fault identification, the RRTNet and DA-LSTM had lower Jaccard values. It attests to the fact that CAR-TATNet guarantees more accurate and dependable EV battery issue diagnosis capabilities.

**Table 8:** Analysis of AUC and Jaccard index.

Classifiers	AUC-ROC	Jaccard index
DA-LSTM	0.9854	0.7183
ATTNet	0.9955	0.7955
RRTNet	0.9895	0.6116
CAR-TATNet	0.9912	0.8587

The robustness of the CAR-TATNet model is further illustrated by the findings of Cohen’s Kappa and Matthews Correlation Coefficient (MCC). Even in the presence of class imbalance, the model achieves the maximum MCC value of 0.91, suggesting a good positive correlation between predicted and actual fault classifications. In addition, when compared to other models, it has a superior Cohen’s Kappa score of 0.9084, as presented in Table 9. The other methods, on the other hand, show lower levels, underscoring their inconsistent fault prediction. The more stable and dependable classification performance of CAR-TATNet is confirmed by the better MCC and Kappa values.

**Table 9:** Analysis of MCC and Cohen’s Kappa.

Classifiers	Matthews correlation coefficient	Cohen’s Kappa
DA-LSTM	0.8081	0.8002
ATTNet	0.8571	0.8522
RRTNet	0.8064	0.8012
CAR-TATNet	0.91	0.9084

**Figure 23:**
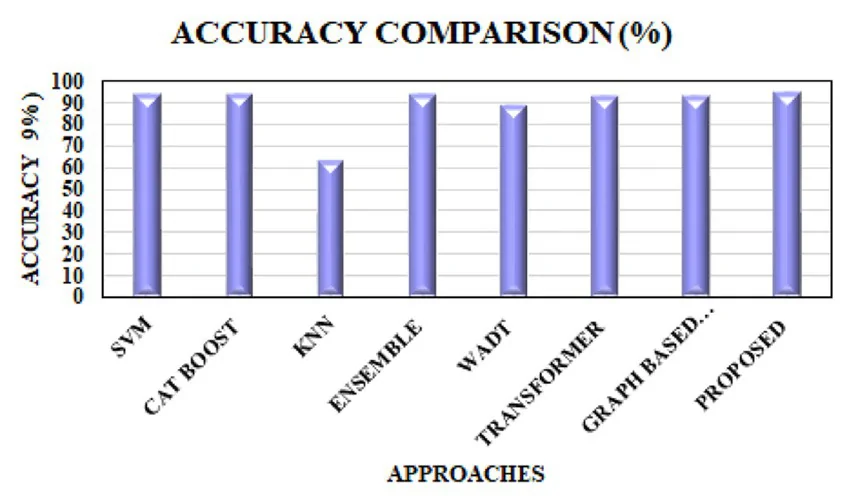
Analysis of accuracy.

The CAR-TATNet model outperforms all other approaches in the accuracy comparison for EV battery defect diagnosis. The SVM (Zhang et al., 2024) and CatBoost (Shah, 2024) have 94% accuracy, while KNN (Thirunavukkarasu et al., 2024) has 63.32%. When compared to individual classifiers, the ensemble technique (Mishra et al., 2024) attains 94%, demonstrating enhanced robustness. The accuracy of the Graph-Based approach (Yao et al., 2023) is 93.2%, while the WADT (Liu et al., 2024) and Transformer (Huang et al., 2024) attain 88 and 92%. It demonstrates that the CAR-TATNet architecture offers enhanced EV battery fault diagnosis capabilities with an accuracy of 94.94%, as displayed in Figure 23.

**Figure 24:**
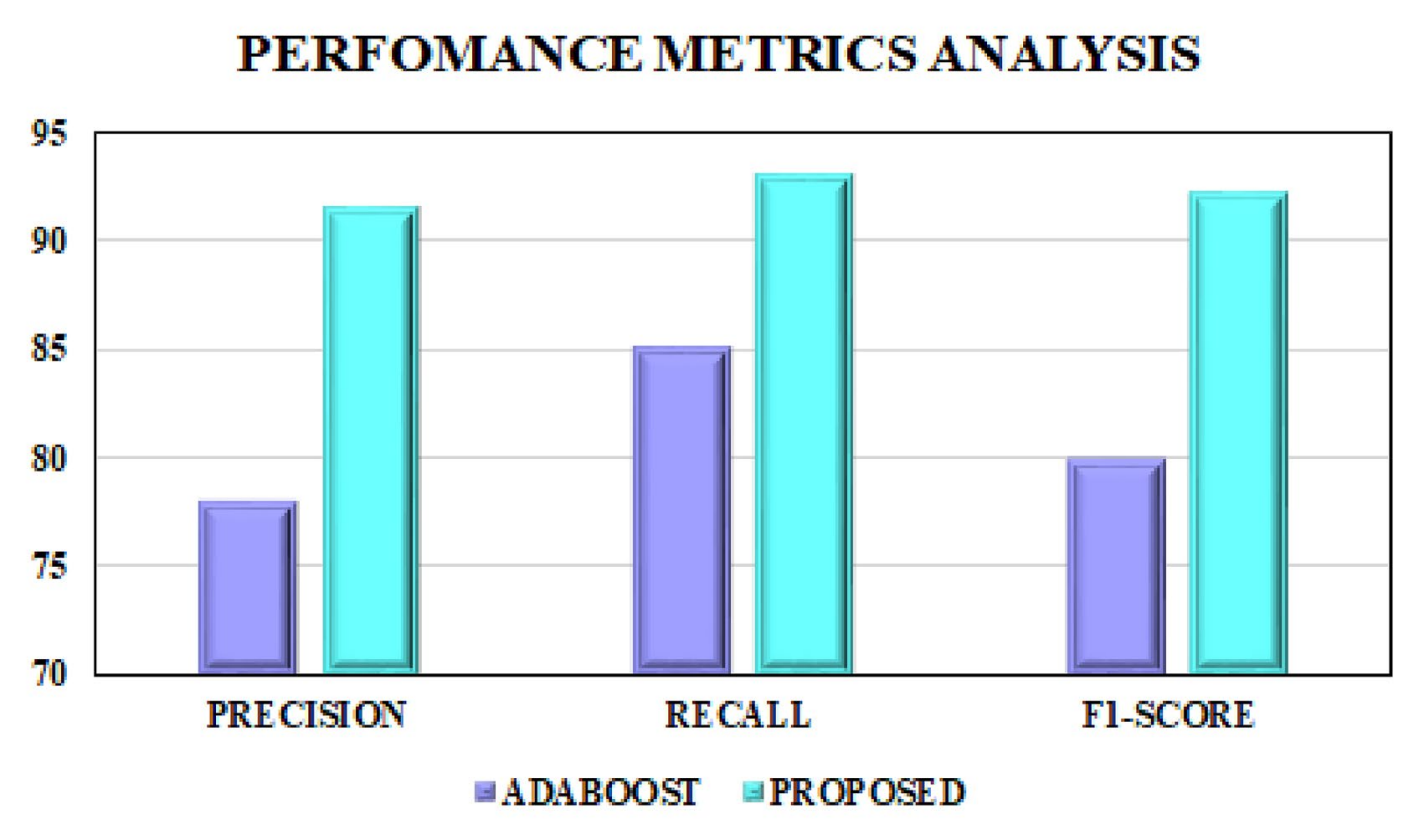
Analysis of performance metrics.

An analysis of performance metrics for the developed approach with Ada boost (Kamboj et al., 2025) approaches are depicted in Figure 24. The developed approach has a better recall than other approaches of 93.08%, indicating the performance of the system is improved.

**Figure 25:**
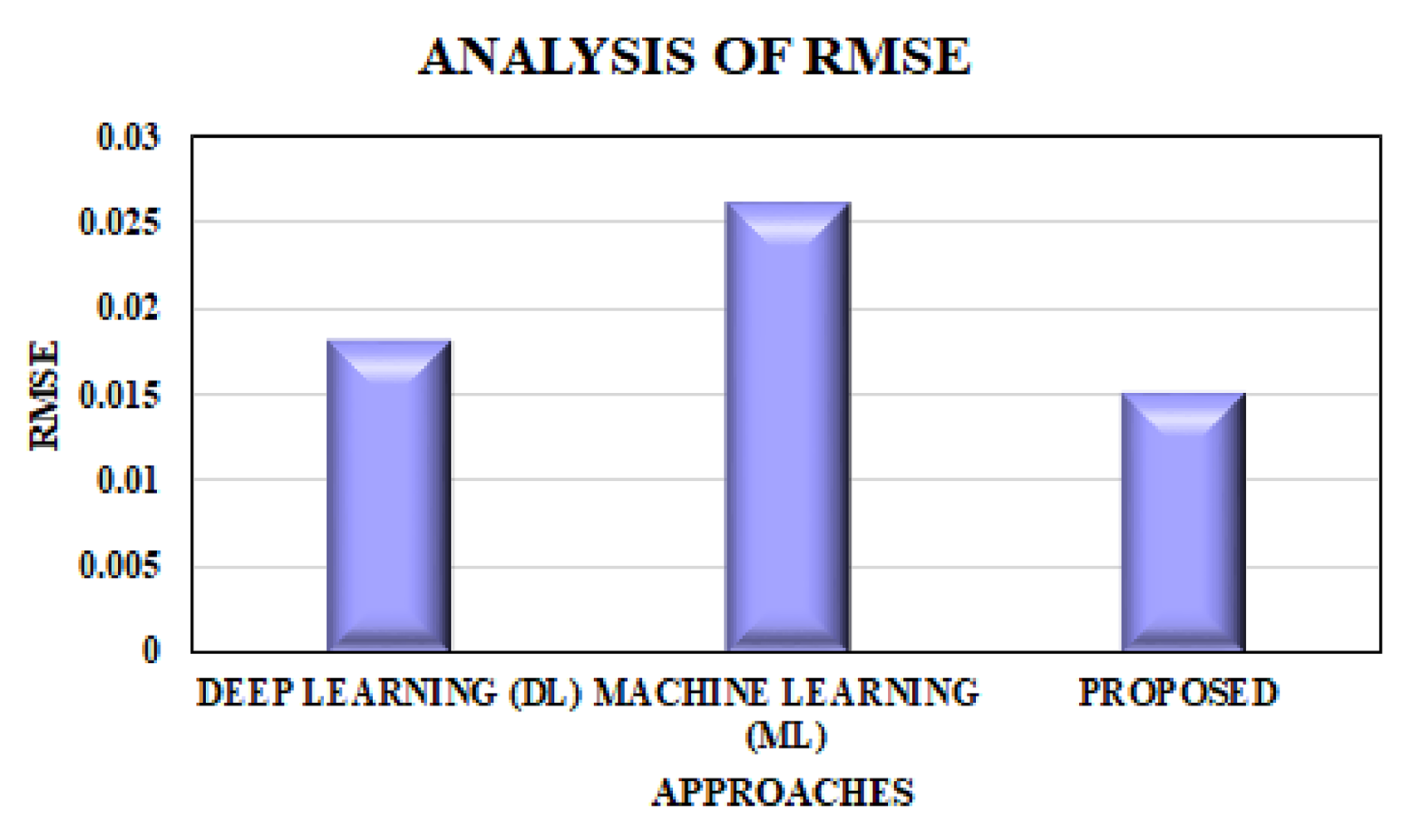
Analysis of RMSE.

The DL (Kaur et al., 2023) and Machine Learning (ML) (Madani et al., 2024) have higher RMSEs of 0.018 and 0.026 than the developed approach with a value of 0.015, as seen in Figure 25, replicating the system’s capability to sustain the durability, protection, and efficacy of the EV battery.

## 5 Conclusion

By combining RBBMO feature selection and CAR-TATNet, this research presents a unique DL-based approach for EV battery fault diagnosis. By utilizing sophisticated preprocessing methods such as ICEEMDAN and STFT for feature extraction, the suggested model successfully tackles the problems of small defect samples and intricate temporal correlations. The model greatly increases its diagnostic accuracy by identifying the most pertinent fault patterns through the use of RBBMO for feature selection. According to experimental results in the Python tool, CAR-TATNet is superior at modelling long-term dependencies in battery operating data and identifying time-varying problem indicators. This strategy outperforms conventional defect detection techniques, indicating its potential for practical EV applications with a superior accuracy of 94.94%. The model is useful for guaranteeing the dependability and safety of EV batteries because of its capacity to adjust to changing operating conditions and identify problems with extreme precision. Future research might concentrate on strengthening the model’s resilience and expanding its applicability to various battery kinds and failure situations.

## Data Availability

The datasets presented in this study can be found in online repositories. The names of the repository/repositories and accession number(s) can be found at: https://drive.google.com/file/d/16ScfAnaG1DRa2kWR1rKbS_bAFpZUDPsm/view?usp=drivesdk.
